# Differing Impacts of Livestock Farming and Ranching on Aquatic Insect Biodiversity: A Global Meta‐Analysis

**DOI:** 10.1111/gcb.70513

**Published:** 2025-09-22

**Authors:** Lindsey A. Barnes, Emily Wenban‐Smith, Grace Skinner, Lynn V. Dicks, Joseph Millard, Andrew J. Bladon

**Affiliations:** ^1^ Department of Zoology University of Cambridge Cambridge UK; ^2^ UK Centre for Ecology and Hydrology Wallingford UK; ^3^ Ecology and Evolutionary Biology Division, Health and Life Sciences Building, School of Biological Sciences University of Reading, Whiteknights Reading UK

**Keywords:** Ephemeroptera, EPT, livestock grazing, macroinvertebrates, Megaloptera, Odonata, Plecoptera, Trichoptera

## Abstract

Recent studies examining global insect biodiversity trends have shown declines for many terrestrial species but increases in some aquatic species, albeit with limited spatial coverage. However, the impact of a wide range of threats on insect biodiversity remains uncertain at a global scale. Livestock farming and ranching constitute approximately 30% of global land use and represent a major and growing threat to biodiversity. Although we know livestock farming and ranching affect aquatic macroinvertebrates via degradation of water quality and habitat, there are no global syntheses of the impacts of livestock on the biodiversity of aquatic insects. Here, we investigate the impact of livestock farming and ranching on the abundance and richness of five major aquatic insect orders: Ephemeroptera (mayflies), Plecoptera (stoneflies), Trichoptera (caddisflies), Megaloptera (dobsonflies and alderflies), and Odonata (dragonflies and damselflies). Our meta‐analysis shows that livestock farming significantly reduces species richness of Ephemeroptera, Trichoptera, and Plecoptera compared to areas with no livestock present. In contrast, we found no overall impact of livestock farming on the abundance of aquatic insects or individual orders, even after accounting for moderators such as livestock type, riparian vegetation presence, and stocking density. The apparent stability in insect abundance, combined with declines in richness, suggests there may be shifts in community composition that cannot be captured with a broad‐scale analysis. Further research is needed at finer taxonomic resolution, coupled with increased reporting of quantitative stocking density and livestock water access, to better understand the apparently heterogeneous effects of livestock on aquatic insects and predict the impacts of further spread and intensification of livestock farming.

## Graphical abstract

This meta‐analysis investigates the impact of livestock farming and ranching on the abundance and richness of major aquatic insect orders. Livestock farming significantly reduced the richness of Ephemeroptera, Plecoptera, and Trichoptera. However, livestock farming did not significantly impact abundance, suggesting finite shifts in aquatic insect community composition. Moreover, this study exemplifies the need for research at finer taxonomic resolutions, as well as increased reporting of quantitative stocking density and livestock water access, to better understand the observed heterogeneous effects of livestock on aquatic insects.

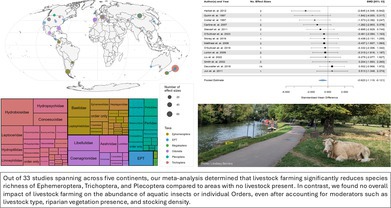

## 1 Introduction

Insects are often the most abundant organisms in freshwater ecosystems and perform essential ecological roles such as litter decomposition, nutrient retention, food sources, and algae removal (Suter II and Cormier 2015, Samways 2020). While aquatic invertebrate communities have been well documented for freshwater quality assessments and as bioindicators of forest health (Sigutová et al. 2019; Das and Maity 2021; Eriksen et al. 2021), global biodiversity trends for aquatic insects are inconsistent. Increasing (Outhwaite et al. 2020; van Klink et al. 2020), decreasing (Baranov et al. 2020; Romero et al. 2021; Rumschlag et al. 2023), and stable (Haase et al. 2023) trends in abundance, richness, and diversity have been reported at regional and global scales. With such a diversity of trends reported, understanding the impact of specific drivers on specific taxa becomes increasingly important. Available information suggests that anthropogenic habitat alteration and the subsequent increase in water pollution are some of the major threats to aquatic insects (Reid et al. 2019; Samways 2020). However, quantifying the impact of threats on insect populations is inherently challenging due to the wide range of anthropogenic pressures and high diversity within Orders (Collen et al. 2012; Wagner 2020).

Developments in global‐scale research have offered new insights into the major threats to aquatic insects. Recently, the International Union for the Conservation of Nature's (IUCN) Red List's comprehensive assessment of Odonata species marked the first global evaluation of extinction risk and threats to an entire insect Order (IUCN 2021, 2024). This allows an assessment of the number of species, globally, that are thought to be impacted by each threat, and therefore the calculation of a simple ranking of threats (Maxwell et al. 2016). In addition, the Global Insect Threat‐Response Synthesis project (GLiTRS; glitrs.ceh.ac.uk), which aims to create an insect threat‐response model using current literature and expert knowledge (Cooke et al. 2025), has run a series of workshops with entomologists to derive the relative importance of threats to insect Orders using the IUCN Red List Threat Classification Scheme (Salafsky et al. 2008; Bladon et al. in prep). Combined threat rankings from the Red List assessments and the GLiTRS expert elicitation workshops have determined livestock farming and ranching to be one of the major threats to Odonata and other aquatic insects. Broader examinations of threats to aquatic systems and invertebrates also highlight the danger posed by runoff from agricultural sources (Collen et al. 2012; O'Callaghan et al. 2019) and identify livestock as a major driver of biodiversity change (IPBES 2019).

The effects of livestock on freshwater ecosystems are well documented. Livestock presence in and around freshwater can cause increases in nutrients (del Rosario et al. 2002; Vidon et al. 2008) and sediment (O'Callaghan et al. 2019), and can lead to a reduction in riparian vegetation and bank stability (Epele and Miserendino 2015; Krall and Roni 2023). Runoff nutrients, sediment, bacteria, and other pollutants are accumulated in lentic systems (e.g., ponds, wetlands), and dispersed downstream in lotic (rivers, streams) systems (Reid et al. 2019). Livestock presence in the surrounding drainage area of a freshwater body (i.e., catchment) may still impact the freshwater ecosystem even when livestock are not adjacent to the sampled habitat (Weijters et al. 2009; Herbst et al. 2012; Larson et al. 2016). Additionally, the impacts of livestock vary depending on the livestock type, grazing management practices, stocking density, and the geological and morphological characteristics of the freshwater body (Matthaei et al. 2006; Herbst et al. 2012; Yoshimura 2012; Holmes et al. 2016). Other direct anthropogenic threats tied to livestock farming and ranching, such as the conversion of forests to grazing pasture, have been shown to affect aquatic insect community assemblage and reduce densities of more sensitive taxa (Quinn et al. 1997; Yoshimura 2012).

Meta‐analyses have become increasingly prevalent in ecological literature and have improved our understanding of anthropogenic impacts on biodiversity worldwide (Gurevitch et al. 2018). Previous meta‐analyses have examined livestock impacts across taxonomic kingdoms (Felton et al. 2010; Dettenmaier et al. 2017; Sartorello et al. 2020; Huaranca et al. 2022), ecosystems (Davidson et al. 2017; Li and Jiang 2021; Schürings et al. 2022; Su et al. 2023), and abiotic properties (Yayneshet and Treydte 2015; Lai and Kumar 2020); however, there are no global meta‐analyses examining the effect of livestock farming and ranching solely on freshwater insects. To address this knowledge gap, we present a quantification of the threat of livestock farming and ranching on the abundance and species richness of five major aquatic insect Orders through a global meta‐analysis, accounting for variation due to livestock intensity, ecological factors (e.g., livestock species, catchment effects, habitat type), and differences between Orders.

Specifically, we ask the following questions:

1. What is the overall impact of livestock on the abundance and richness of major Orders of aquatic insects (Ephemeroptera, Trichoptera, Plecoptera, Odonata, Megaloptera)?
2. Do the impacts of livestock farming and ranching differ between aquatic insect Orders?
3. Do higher intensities or densities of livestock grazing have a greater impact on the abundance or species richness of aquatic insects than lower intensities?

## 2 Methods

### 2.1 Literature Search

The threat of livestock farming and ranching was defined using the IUCN Red List Threat Classification Scheme (Version 3.3 (Salafsky et al. 2008)), a recognized and expert‐reviewed schema. To facilitate the initial literature search, we used the PICO framework (Richardson et al. 1995) to formulate a main research question (Table 1). Using the PICO research question as a guideline, the initial search string below was created (formatted for Scopus) to extract peer‐reviewed articles examining the impacts of livestock farming and ranching on aquatic insects anywhere in the world:

**TABLE 1:** Description of the main research question structured using the PICO framework.

Criteria	Definition
Population	Any species of Odonata, Ephemeroptera, Plecoptera, Trichoptera, or Megaloptera
Intervention	Areas impacted by threats falling within the definition of IUCN Red List Threat 2.3—Livestock farming and ranching; any presence of livestock, including sites impacted by deforestation for livestock grazing/pasture use
Comparison	Low or absent livestock farming and ranching
Outcome	Abundance, species richness, and diversity

TITLE‐ABS‐KEY ((odonat* OR dragonfl* OR damselfl* OR “freshwater *invertebrate$”) AND (livestock OR ranch* OR farm* OR graz*) AND (abundance OR richness OR diversity))

We tested the suitability of the search string by assessing the relevance to the PICO question of 20 randomly selected titles and abstracts returned from the search. Using the R package *litsearchr* (Grames et al. 2019), we identified common terms between relevant results to include in subsequent search strings. Common terms between non‐relevant results were either removed or explicitly stated to exclude them within the updated search strings. We repeated this process until the revised search strings no longer returned new, relevant results. We conducted the systematic literature search using the final strings on 1 December 2023 on the platforms Scopus and Web of Science, using no filters or database exclusions, and included results in all languages and publication years (Table S1). The results retrieved from both platforms were combined and had duplicate results removed immediately, using the *litsearchr* function “remove_duplicates” and through later manual checks of the returned papers (*n* = 1164).

To screen the deduplicated results for eligible papers, we followed PRISMA guidelines (Figure S1, (Page et al. 2021)) and the meta‐protocol outlined by Millard et al. (2023), which included preregistration and the upload of a systematic literature screening and data extraction protocol (Barnes and Bladon 2023, *see* https://osf.io/e39fz). Returned studies were screened in two rounds: title and abstract, then full text. For each round of screening, two reviewers (L.A.B. and E.W.‐S.) both screened the same subset of returned papers (50 publications for title and abstract, 10 publications for full text) following the pre‐established protocol to ensure repeatability. Differences in inclusion were discussed and the protocol clarified where necessary before screening continued. After addressing comments from the independent reviewer, the final screening criteria were used for all papers (Table 2).

**TABLE 2:** Overview of the final screening criteria used for the exclusion of studies not relevant to the PICO question (Table 1).

First round, title and abstract	Second round, full text
No taxa of aquatic insect examined Livestock presence or impact was not examined Abundance, richness, and/or diversity was not examined Study was not in freshwater	Could not determine livestock impacts due to other disturbance, and/or study does not provide details of livestock presence or use of treatment pasture for livestock Publication was not peer‐reviewed or empirical Study did not examine biodiversity metrics of aquatic insects Study examined impacts of feral or wild species Full text was unavailable

As part of the pre‐established criteria, we excluded publications that were not peer‐reviewed (e.g., book chapters, dissertations) and literature that did not follow an experimental or quasi‐experimental approach (e.g., literature reviews, taxonomic recordings of species). Publications in languages other than English were included and translated to English using the free translation website DeepL (DeepL 2024). For publications that had their full text screened (*n* = 311), we recorded the specific reason for exclusion for each study (Table 2, Figure S1). During full‐text screening, a subset of publications (*n* = 54) met all research criteria but did not present data in a format that could be used for a meta‐analysis (e.g., no error estimates, results only presented via linear dimensionality visualizations like Principal Component Analyses). For these papers, we contacted the authors to request raw data or additional information and excluded papers where we did not hear back (Figure S1).

### 2.2 Data Extraction

From the 33 publications which passed full‐text screening, we extracted data directly from text, tables, and figures (figures via WebPlot Digitizer (Rohatgi 2024)). Core data were collected on the insect taxon studied, sample size, estimated mean biodiversity metric (abundance, species richness, or diversity), and estimated error (as confidence intervals, standard deviation, or standard error) for sites with livestock present (treatment) and sites with low or no livestock presence (control). All extracted data, including publication details (i.e., journal name, language) and experimental metadata (i.e., study site coordinates), were entered into a standardized data extraction sheet structured following recommendations from the ecological meta‐analysis platform Dynameta (Skinner et al. 2023). We extracted data for the finest taxonomic level available (e.g., genus or species), although in some cases, data were coarse and only provided for multiple Orders combined (i.e., Ephemeroptera, Plecoptera, and Trichoptera (EPT)). If biodiversity metrics were provided without error estimates and could not be averaged by site or year, we averaged raw data or mean estimates across the finest recorded taxonomic level to obtain a suitable error calculation at a courser taxonomic resolution.

Based on prior research examining livestock impacts on freshwater habitats and macroinvertebrates, commonly reported information was extracted as potential moderators whenever reported in a study (Table 3). These moderators were: the livestock species present at the grazed site (Matthaei et al. 2006; Bilotta et al. 2007; McDowell and Wilcock 2008); the scale of the treatment‐control comparison site level or catchment level grazing and exclusion; (Weijters et al. 2009, Herbst et al. 2012); the habitat type at the control site forest or grassland; (Quinn et al. 1997, Yoshimura 2012); and the life stage (adults or larvae) of the collected insects (based on whether the survey method was aquatic or terrestrial if not explicitly reported (Smith et al. 2002, Petersen et al. 2004)). However, other environmental information, such as stream habitat (e.g., riffles, pools), seasons (e.g., autumn, spring), or collection year, were pooled together to create a more comparable dataset between studies, as reporting on these factors individually was inconsistent between publications.

**TABLE 3:** List of all moderators included as fixed effects for meta‐analysis multi‐level modeling. Categorical moderators are listed with all included levels.

Moderator	Description	Levels/unit
Livestock type	Broad groups of livestock types reported in studies, with mixed groups of livestock combined with unreported (unknown) livestock species	Cattle Cattle & Sheep Mixed/Other/Unknown
Study scale	Scale of research: either site‐level (e.g., cattle exclosures, stream reaches) or catchment‐level (entire drainage area)	Catchment‐level Site‐level
Control vegetation	Main vegetation around control sites; for catchment‐scale studies, also includes the dominant vegetation within the catchment	Grassland Forest
Life stage	Assumed life stage of surveyed taxa based on study description and/or sampling methods. If not explicit, “larva” assigned to aquatic collection methods (e.g., Surber samplers) and “adult” assigned to terrestrial methods (e.g., light traps, malaise traps)	3 Adult 4 Larva
Intensity	Qualitative values of livestock intensity reported in individual studies for treatment and control sites (high, medium, low, none). Categories created using combinations of study reported intensities (Treatment Intensity/Control Intensity)	Low/None High/Low High/None Med/Low
Density	Quantitative value of stocking density, transformed into Animal Unit Equivalents (AUE)/hectare for standardization (Table S2)	AUE per hectare

Where possible, we additionally recorded information on categorical and numerical grazing intensity. If studies explicitly reported “low”, “medium” or “high” livestock presence in either the treatment or control site, it was recorded as such. Different grazing practices within a study were also assigned a categorical level of livestock intensity, based on the information provided by the authors (e.g., a publication may suggest year‐round grazing would be “high” intensity, whereas rotational grazing would be “low” intensity). These publication‐defined categorical intensities for the treatment and control sites were combined into paired treatment/control categories for analysis; for example, “high” livestock presence at a treatment site and “low” livestock presence at a control site would be categorized as a “High/Low” livestock intensity comparison (Table 3).

Numerical stocking densities were often reported in papers as a single unit of animals per hectare. When stocking density for a single site was provided as a range, the maximum value was recorded as we assumed that the maximum stocking density provided related to the maximum possible threat imposed on the site. However, if stocking densities were reported across multiple sites that needed to be averaged to calculate an error estimate, the average stocking density across all pooled sites was recorded. To standardize stocking density of varying units and livestock types, we converted all recorded stocking density units to Animal Unit Equivalents (AUE (Ogle and Brazee 2009, Most and Yates 2022)) per hectare (Table S2).

## 3 Data Analysis

Despite being explicitly searched for, only one paper reported a measure of species diversity. Therefore, analyses were conducted only examining livestock effects on species richness and abundance. All statistical analyses were conducted in R version 4.3.1 (R Core Team 2023) using the *metafor* package (Viechtbauer 2010a). All error estimations reported as standard error or 95% confidence intervals were converted to standard deviation prior to effect size calculation. For each observation, effect sizes (Hedges' g) were calculated using the means and standard deviations for each control and treatment pair, corrected for bias toward small sample sizes (Hedges 1981). Effect sizes were examined using a linear mixed effects model framework, estimated using restricted maximum likelihood (REML), and fitted with a random intercept of observation nested within study to account for similarity between observations originating from the same paper. Models were fitted using the ‘rma.mv’ function from the *metafor* package, following recommended settings (e.g., use of t‐distributions for model coefficient estimates (Viechtbauer 2010b)). This basic model structure was applied to all models.

Due to the acceptance of publications reporting on either complete absence or “low” intensities of livestock grazing in control sites, we ran a basic linear mixed effects model testing for differences in effect size between these two control subgroups before running any model addressing the main research questions. As no study of species richness reported low livestock density as a control, the subgroup analysis of “control type” (complete absence of livestock versus low density presence of livestock) was only fitted for abundance data. This test for subgroup differences between control sites indicated there was no significant effect (*Q*_M_ = 2.56, *p* = 0.126) on aquatic insect abundance (Figure S2). Despite many observations for controls of low livestock intensity (*n* = 143), these originated from only three studies, which is suggested to be too low to extract true differences between subgroups (Valentine et al. 2010). Therefore, we included studies with low presence of livestock as controls with other studies reporting controls as complete absence of livestock in all further abundance analyses.

Eight separate models were fitted to assess the impacts of livestock farming and ranching on aquatic insect abundance and richness. Of these, six models assessed general livestock impacts on all aquatic insect richness and abundance and the abundance of the four most reported Orders (Ephemeroptera, Trichoptera, Plecoptera, and Odonata; see Sections 3.1 and 3.2). The remaining two models assessed the impacts of categorical livestock intensity and quantitative stocking density, respectively (see Section 3.3). Due to limited effect sizes for species richness, only abundance was examined for the models on individual Orders and livestock intensity and density.

### 3.1 What Is the Overall Impact of Livestock on the Abundance and Richness of Major Orders of Aquatic Insects?

To assess the overall impacts of livestock presence on aquatic insects, one random effects model using the basic structure outlined previously was fitted to all richness observations due to the limited number of studies and observations. In contrast, there was a large sample size for abundance; therefore, differences due to livestock type, study scale, life stage, and type of control site could be assessed (Table 3).

We attempted to include these moderators as fixed effects within a single initial maximal model; however, we did not include moderators and their two‐way interactions if the sample size was 20 or fewer effect sizes per grouping, based on a modification of the standard, yet arbitrary “ten events per one variable” rule of thumb (Harrell Jr. et al. 1996; Steyerberg et al. 2000). The final maximal model for all abundance observations contained fixed effects of livestock type, control riparian vegetation (forest or grassland), study scale (site/reach or catchment), and life stage (adult or larvae), and the two‐way interactions between riparian vegetation and livestock type, and between riparian vegetation and study scale. These interactions were included based on previous literature reporting variations in water quality and insect communities depending on the possible antagonistic and synergistic influences of these factors (Quinn et al. 1997; Bilotta et al. 2007; Herbst et al. 2012; Yoshimura 2012; Faria et al. 2021).

Collinearity of all included categorical moderators was assessed by determining the Variance Inflation Factor (VIF). If VIF was 10 or above, moderators were reviewed individually using chi‐squared tests, as VIF values can be influenced by other factors than just collinearity (O'Brien 2007). Ultimately, no moderator was removed based on VIF, as no chi‐squared test determined moderators with high VIF to be significantly correlated.

As REML‐fitted models assume the correct structure of fixed effects, it is generally recommended they be re‐fitted to a maximum likelihood (ML) estimation before performing information‐criterion based model selection (Viechtbauer 2010a). Therefore, once any potential issues with collinearity were resolved, each resulting maximal model was run using maximum likelihood (ML) estimation. Corrected Akaike Information Criterion (AIC_C_) model selection was performed on each maximal model using the `dredge` function in the R package *MuMIn* (version 1.48.4 (Bartoń 2024)). If multiple models were within two ΔAIC_C_ of the model with the lowest AIC_C_, the most parsimonious model was chosen as the final model. The final models had the formula extracted and were re‐run with REML to correct for bias in parameter estimates when using ML.

### 3.2 Do the Impacts of Livestock Farming and Ranching Differ Between Aquatic Insect Orders?

To assess the overall impacts of livestock presence on the abundance of the four best‐represented Orders of aquatic insects (Ephemeroptera, Trichoptera, Plecoptera, and Odonata), the same maximal model was fitted with the abundance of each Order as the response variable. Following the methods for the full abundance model, each moderator's sample size was assessed on a model‐by‐model basis and removed if there were fewer than 20 effect sizes per group. Similarly, VIF was examined for all moderators in the final maximal Order‐specific model before determining the final model through AIC_c_ model selection.

No moderator fitted to an Order‐specific model was removed due to collinearity issues; however, maximal models for each Order differed due to sample size variability. For both Ephemeroptera and Plecoptera, the maximal models included fixed effects of study scale and control riparian vegetation. For Trichoptera, the moderators of control riparian vegetation, study scale, livestock type, life stage, and the interaction between riparian vegetation and study scale were fitted to the maximal model. Lastly, the maximal model for Odonata included the control riparian vegetation, study scale, and life stage moderators.

### 3.3 Do Higher Intensities or Densities of Livestock Grazing Have a Greater Impact on the Abundance of Aquatic Insects Than Lower Intensities/Densities?

We examined the effects of livestock intensity and density on aquatic insect abundance by fitting two independent models: one for all records of qualitative livestock intensity and one for all quantitative records of stocking density, with only “intensity” or “density” fitted as moderators, respectively (Table 3). No other moderators were fitted to these models due to the smaller number of effect sizes and fewer studies where intensity/density were reported; therefore, each model was assumed to have the correct moderator formula and was fitted with REML.

### 3.4 Model Fit and Publication Bias

Publication bias is an intrinsic problem within meta‐analyses, as studies reporting significant results have a greater probability of publication than those reporting non‐significant results, which can also manifest in studies omitting non‐significant results entirely. We examined publication bias for each model using a combination of funnel plots and Egger's test, a regression analysis on the effect sizes and their standard deviations (Egger et al. 1997).

Following the guidelines of Nakagawa and Santos (2012) for biological meta‐analyses and Viechtbauer's (2021) discussions on assessing meta‐analysis model fit, we report heterogeneity using the *I* ^2^ statistic, an estimation of the variation between studies not due to sampling error. *I* ^2^ values were interpreted following general thresholds suggested by Deeks et al. (2013): 0% to 30%: possibly unimportant; 30% to 50%: possible moderate heterogeneity; 50% to 75%: possible substantial heterogeneity; 75% to 100%: likely considerable heterogeneity.

To assess model fit, we evaluated model normality using quantile‐quantile (QQ) plots and identified outliers using Cook's distance analysis (Cook 1977). Any observations above the outlier threshold (determined using the *metafor* function “cooks.distance” (Viechtbauer and Cheung 2010)) were examined further for potential data entry or calculation errors, but all outliers were ultimately considered to reflect natural variation among the studies' sample populations and were retained in the analysis.

As no meta‐analysis outcome measure fully meets all assumptions (Viechtbauer 2021), interpretations of effect sizes were made with the above evaluations in mind, especially when heterogeneity or publication bias were considered to be significant. For general interpretation of effect sizes, negative estimates of Hedges' *g* were interpreted to suggest declines in the assessed biodiversity metric due to the presence of livestock, and positive estimates were interpreted to indicate an increase.

## 4 Results

### 4.1 Data Composition

**FIGURE 1:**
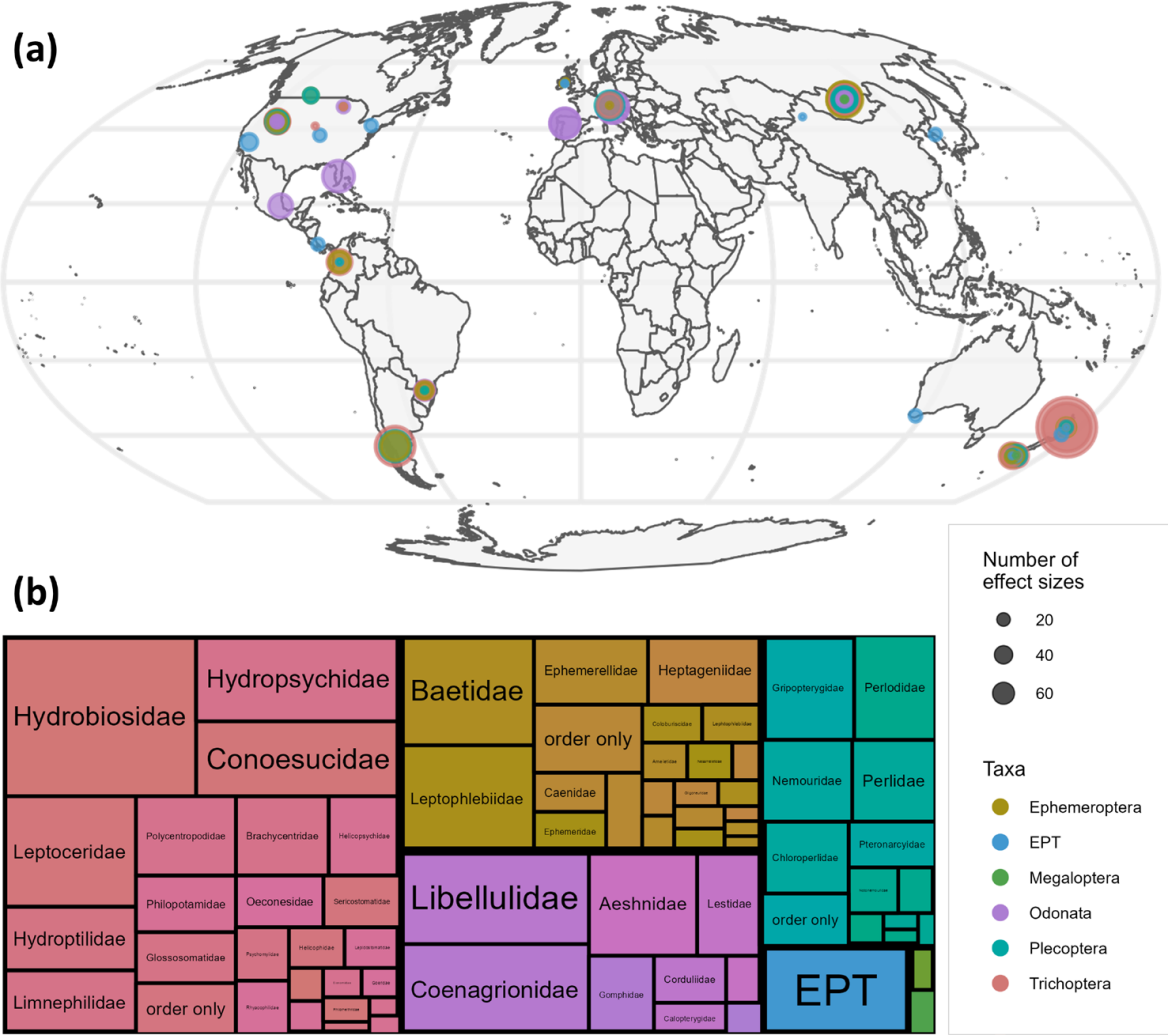
Geographic locations and taxonomic representation of all 33 studies and 656 observations of effect sizes included in analyses. (a) The location of all observations (circles) colored by taxonomic Order (Ephemeroptera, Plecoptera, Trichoptera, Megaloptera, Odonata) or Subclass (combined EPT). The circle circumference represents the number of effect sizes per Order, with a larger circumference indicating more effect sizes. Map lines delineate study areas and do not necessarily depict accepted national boundaries. (b) Tree plot of the taxonomic resolution of observations, colored by taxonomic Order or Subclass. The area of the boxes represents the number of observations per group, and boxes of the same color show the distribution of families (if applicable) within each Order.

Thirty‐three (33) studies, published from 1992 to 2023, were included in analyses after full‐text screening and author correspondence (Table S3). Studies were conducted in 15 countries across five continents, with most occurring in New Zealand (*n* = 10, 30.3%) and the United States (*n* = 7, 21.2%; Figure 1). From these studies, 656 effect sizes (ranging from one to 111 per study) were calculated for abundance (*n* = 622) and richness (*n* = 34). All five orders of aquatic insects were represented, with Trichoptera (*n* = 280, 42.7%) and Ephemeroptera (*n* = 138, 21.0%) being the most commonly reported taxa (Figure 1). In contrast, Megaloptera accounted for only 0.6% of observations (*n* = 4). Pre‐examination of publication bias showed that 20% of studies (*n* = 7) reported biodiversity metrics only for taxa for which there was a significant effect.

### 4.2 What Is the Overall Impact of Livestock on the Abundance and Richness of Major Orders of Aquatic Insects?

**FIGURE 2:**
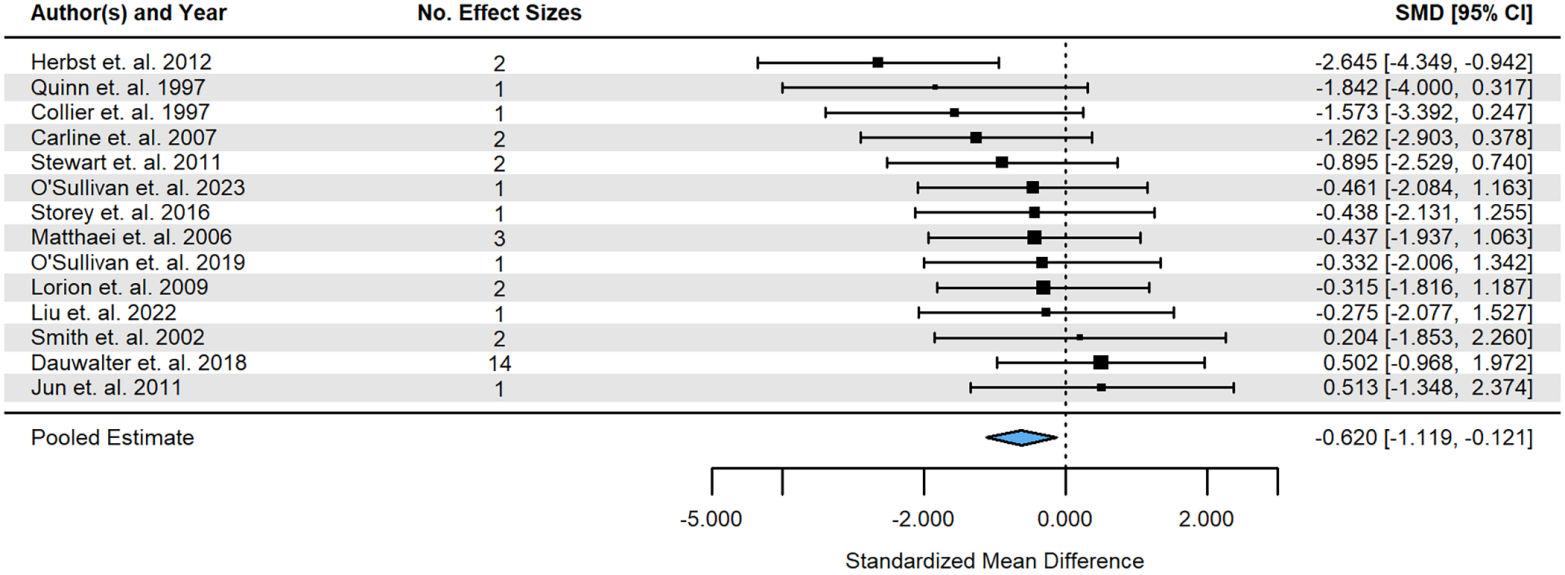
Forest plot of intercept‐only multilevel model for the effect of the presence of livestock farming and ranching on the richness of three aquatic insect Orders: Ephemeroptera, Plecoptera, and Trichoptera (EPT). The overall mean estimate is depicted by the blue diamond at the bottom of the plot, which suggests presence of livestock farming is associated with a significant decrease in EPT richness. Analysis was conducted on individual effect sizes (corrected standardised mean difference, Hedges' *g*; *k* = 34), however the figure shows estimates aggregated by study (*n* = 14). Black squares represent the pooled estimates for each study, with the size of the square indicating the number of effect sizes. Confidence intervals for each study are depicted with horizonal black lines.

From the 14 studies which reported species richness, 34 observations were obtained for three aquatic insect taxa: Ephemeroptera, Plecoptera, and Trichoptera. Reports of combined EPT accounted for 50.0% of all richness observations. The presence of livestock caused a reduction in EPT richness (Estimate ± SE = −0.620 ± 0.231, 95% CI = [−1.119, −0.121]; *p* = 0.019; *I* ^2^ = 81.72%; Figure 2). No significant publication bias was found from the funnel plots (Figure S3) or Egger's test (Table S4; *p* = 0.086, *z* = 1.714).

### 4.3 What Is the Overall Impact of Livestock on Aquatic Insect Abundance?

For aquatic insect abundance, 622 observations across 27 studies represented all five major aquatic insect Orders and combined EPT, with the most reported taxa being Trichoptera (*n* = 272, 43.7%), Ephemeroptera (*n* = 133, 21.4%), Odonata (*n* = 117, 18.8%), and Plecoptera (*n* = 91, 14.6%). The best fitting model chosen by AIC_c_ included no fitted parameters and found no impact of livestock grazing on aquatic insect abundance (estimate ± SE = −0.129 ± 0.103, 95% CI = [−0.341, 0.083], *p* = 0.222; *I* ^2^ = 50.50%; Figure S4 and Table S5). The model was not significantly affected by publication bias, according to the funnel plot (Figure S3) and Egger's test (Table S4, *p* = 0.204, *z* = −1.269).

### 4.4 Do the Impacts of Livestock Farming and Ranching Differ Between Aquatic Insect Orders?

The final models selected by AIC_c_ for Ephemeroptera (Table S6), Trichoptera (Table S7), and Odonata (Table S8) retained no fitted moderators and found no effect of livestock on the abundance of any of these three orders. In contrast, study scale was retained in the final model for Plecoptera (Table S9), suggesting a marginal reduction in Plecoptera abundance when livestock were present at the catchment level (estimate ± SE = −0.381 ± 0.181, 95% CI = [−0.791, 0.028], *p* = 0.064) and higher Plecoptera abundance when livestock was present at a site level (estimate ± SE = 0.616 ± 0.259, 95% CI = [0.029, 1.202], *p* = 0.042; *I* ^2^ = 37.07%). There was no evidence for publication bias in the Ephemeroptera and Trichoptera models, but Egger's test indicated a significant possibility of publication bias for the Plecoptera (*p* = 0.019, *z* = −2.351) and Odonata (*p* = 0.027, *z* = −2.212) models (Table S4).

### 4.5 Do Higher Intensities or Densities of Livestock Grazing Have a Greater Impact on the Abundance of Aquatic Insects Than Lower Intensities or Densities?

Abundance observations with categorical livestock intensities were examined for 207 effect sizes across six publications. All five aquatic insect orders were represented in the model, with the most effect sizes recorded for Odonata (*n* = 74) and the fewest for Megaloptera (*n* = 2). There was no significant difference in aquatic insect abundance between any categories of livestock intensity (Table S10); however, model heterogeneity was high (*I* ^2^ = 82.88%), an indication of high variance between studies. Additionally, Egger's test indicated a significant possibility of publication bias (*p* = < 0.0001, *z* = −4.574; Table S4).

Numerical livestock densities were reported for 48 effect sizes across five publications for Orders Ephemeroptera, Odonata, Plecoptera, Trichoptera, and combined EPT. There was no effect of livestock stocking density on aquatic insect abundance (estimate ± SE = −0.039 ± 0.193, 95% CI = [−0.427, 0.348], *p* = 0.839; *I* ^2^ = < 0.001%; Figure S5). No significant publication bias was suggested by the funnel plots (Figure S3) or Egger's test (*p* = 0.382, −0.872; Table S4) for the density model.

## 5 Discussion

Through a global meta‐analysis, we found that the species richness of aquatic insects was lower at sites grazed by livestock than at ungrazed sites. However, there was no difference in overall abundance or the abundance of individual Orders between grazed and ungrazed sites, except for Plecoptera. Sites with livestock present at local scales had a higher abundance of Plecoptera than sites without livestock, but freshwater catchments with livestock present had a marginally lower abundance of Plecoptera than catchments without livestock. There was no other effect of livestock type, stocking density, insect life stage, or riparian vegetation on any other metric of insect abundance (Table S10).

### 5.1 What Is the Overall Impact of Livestock on the Richness and Abundance of Major Aquatic Insect Orders?

Unlike recent literature reviews providing summaries of livestock impacts on aquatic insect biodiversity (O'Callaghan et al. 2019; Krall and Roni 2023), our results indicated that livestock farming and ranching reduce species richness of Ephemeroptera, Plecoptera, and Trichoptera (Figure 2). However, these findings align with other large‐scale studies that have reported declines in aquatic insect richness in response to agriculture and other anthropogenic land‐use changes (Epele and Miserendino 2015; Schürings et al. 2022; Rumschlag et al. 2023). In our richness analysis, the substantial heterogeneity (*I* ^2^ = 81.72%) suggests that livestock presence alone does not explain the variance between studies, but limitations in sample size (*k* = 34) prevented any additional exploration through the addition of fixed effects (Deeks et al. 2013). Previous studies have shown significant declines in EPT richness due to increased fine sediments (Matthaei et al. 2006; Beermann et al. 2018), which can be directly caused by livestock‐mediated erosion of stream and pond banks and disturbance of the benthic zone (Vidon et al. 2008). Other studies have reported richness declines due to elevated water salinity levels (Beermann et al. 2018), which can be a result of other agricultural practices and irrigation (Pulido‐Bosch et al. 2018). Ultimately, additional research is needed to better understand the mechanisms causing the reduction of EPT richness associated with livestock farming.

Broadly, the differing patterns observed for abundance and richness suggest that the presence of livestock may influence aquatic insect biodiversity at finer taxonomic resolutions than broad assessments at an Order‐level scale can discern. The stable abundance observed alongside reduced richness suggests shifts in community composition that will only be detectable via examinations of functional groups or lower taxonomic levels. Indeed, in an examination of Patagonian wetlands, Epele and Miserendino (2015) found that research on finer taxonomic resolutions of aquatic insects provides more accurate results and a better reflection of the impacts of livestock and other anthropogenic disturbances. As a testament to this, Kaboré et al. (2016) observed changes in macroinvertebrate abundances, specifically noting community composition changes due to likely increases of sediment and organic nutrients from livestock presence. Our results hint at a similar trend, but more field data are needed to be able to quantify changes at a higher taxonomic resolution.

### 5.2 Do the Impacts of Livestock Farming and Ranching Differ Between Aquatic Insect Orders?

Three of the four Order‐specific models did not retain any environmental moderators, suggesting that neither livestock presence nor the interaction of livestock with any environmental factor had a significant global impact on populations of Trichoptera, Ephemeroptera, and Odonata. Only our Plecoptera model retained the moderator of study scale, suggesting the abundance of Plecoptera is higher where livestock are present at a local, reach scale, compared to when livestock are only present at a broader catchment level. This finding is unexpected given the known sensitivity of stoneflies to anthropogenic pollutants (Pond 2012; Brand and Miserendino 2015; Eriksen et al. 2021). Furthermore, it would be expected that biodiversity trends observed for livestock presence at site levels would be reflected, albeit diminished, in studies examining presence at a larger catchment level. Despite this, a marginal negative effect of on livestock on Plecoptera abundance for catchment scale observations was reported in our model.

It is possible this result stems from issues within the dataset, as the model had a high probability of publication bias based on Egger's test. Therefore, our result may reflect high methodological and/or ecological variance between the parent studies rather than a true effect on Plecoptera abundance. Outlier checks identified at least two observations with high potential to skew results, and these studies only reported significant results, likely contributing to the positive estimate for Plecoptera abundance (Afonso et al. 2024).

Unfortunately, there are few studies that explicitly examine Plecoptera biodiversity metrics in response to anthropogenic disturbances that can be used as external comparison, as many Order‐level examinations are combined with Trichoptera and Ephemeroptera. One study specifically examining Plecoptera and Trichoptera found richness to better reflect anthropogenic disturbances than Order‐level abundance assessments (Pond 2012), thus highlighting the potential limitations of Order‐specific abundance evaluations that are not able to assess changes in community assemblage. Without such information, additional research on livestock impacts on Plecoptera abundance is needed to explore the validity of the result found here.

While non‐significant, the close‐to‐neutral estimate for Odonata abundance (Table S10) between grazed and ungrazed sites may suggest a balance between detrimental and beneficial effects of livestock on this Order. Although Odonata are known to be negatively affected by livestock‐induced increases in suspended sediment and destruction of riparian vegetation, man‐made livestock watering structures (e.g., ponds, troughs) can serve as alternative habitat (de Paz et al. 2020). Additionally, dragonflies (suborder Anisoptera) have been reported to frequent disturbed habitats, such as livestock pastures, more than their damselfly (suborder Zygoptera) or EPT counterparts due to their greater dispersal ability and dependence on sunlight for thermal regulation (Oliveira‐ Junior et al. 2017). Regardless, the influence of significant publication bias in this model necessitates further examination of the stability of Odonata abundance in regard to livestock presence.

### 5.3 Do Higher Intensities or Densities of Livestock Grazing Have a Greater Impact on the Abundance of Aquatic Insects Than Lower Intensities or Densities?

Analyses of both categorical stocking intensity and quantitative stocking density of livestock found no significant effects on aquatic insect abundance, in contrast to previous studies reporting significant negative impacts of stocking density on aquatic insect biodiversity (Braccia and Voshell 2007; McIver and McInnis 2007; Epele and Miserendino 2015). Our findings were constrained by a limited number of studies and observations, as both analyses were conducted on fewer than 10 studies. Additionally, the subjective categories of livestock intensity likely limited comparability between studies that report such categorical assignments, as there is no way to test author consensus of categorical levels between independent publications. Similar issues for categorical stocking intensity were reported from a recent meta‐analysis of livestock impacts on plants and terrestrial consumers (Huaranca et al. 2022). The authors called for researchers to report quantitative stocking density whenever possible, as over half of their included studies did not report a measurement. We found the same problem for studies on aquatic insects, as only six studies (18%) included in this meta‐analysis reported a numerical measurement of stocking density. Without a repeatable numerical quantification, it is difficult for researchers to determine a general level of exposure where aquatic insects are most negatively impacted by livestock presence.

Due to the limited reporting of stocking rate, we could not include moderators such as riparian vegetation and livestock type in the intensity and density models. This leaves another severe knowledge gap when attempting to quantify the impacts of stocking density on aquatic insects. Herbst et al. (2012) emphasized the distinction between stocking rates and direct grazing‐related disturbances: although stocking rates can indicate general exposure levels, they do not accurately reflect actual grazing disturbances, such as direct livestock water access, which can vary depending on local stream conditions and species' grazing patterns. This highlights an important gap in the literature for future research to address.

## 6 Limitations and Knowledge Gaps

### 6.1 Inconsistent Reporting of Livestock Management Practices

We attempted to account for different livestock management practices, but publications varied widely in the level of detail provided, which increased the complexity of the analysis. Management practices and stocking rates were sometimes reported, but often were only recorded as general livestock presence, mostly due to difficulties in obtaining detailed information on management practices and stocking rates on private land. Studies which did report grazing management were highly variable, including practices such as rotational grazing, traditional nomadic herding, and patch‐burn grazing. While we attempted to categorize these management practices into intensities based on publication descriptions, this approach obscured any differences between the practices, which will have added noise to the data. Moreover, a detailed analysis on livestock management practices was not feasible due to the limited number of studies which reported such information and the relatively high number of different management practices.

A larger limitation was the inconsistent reporting on whether livestock were allowed to enter waterbodies. Freshwater with unrestricted livestock access has been shown to experience substantial ecological degradation, including reduced riparian vegetation and bank stability, in addition to declines in general water quality (Strand and Merritt 1999; Conroy et al. 2016; O'Callaghan et al. 2019). Numerous studies examining the removal of livestock from riparian areas and waterbodies have reported beneficial effects across aquatic communities and bank vegetation (Herbst et al. 2012; Holmes et al. 2016; Poessel et al. 2020; Krall and Roni 2023). However, 30% of our included studies did not report livestock waterbody access, which hindered our ability to fully assess the nature of livestock presence and the subsequent impacts on aquatic insect communities. This underscores the need for more detailed reporting of livestock management practices in future research to tease apart context‐dependent effects.

Alongside differences in livestock management practices, some grazing lands were routinely managed, adding to the variation between studies. While many pastures were created from clearing forests and other vegetation, “improved” pastures were created by either the addition of fertilizers (Steinman et al. 2003) or seeding with non‐native grasses to increase forage (Quinn et al. 1992, 1997; Scott et al. 1994; Melo et al. 2003). Seeding of exotic forage introduces an additional threat of exotic vegetation to adjacent aquatic insect communities and was almost exclusively seen for research conducted in New Zealand. Previous research has shown decreases in macroinvertebrate biodiversity and changes in functional feeding group assemblage due to the presence of exotic riparian vegetation (Clarke et al. 2004; Ceilley et al. 2005). Moreover, specific research in New Zealand, conducted across 88 rivers, showed significant impacts on aquatic macroinvertebrate community structure, diversity, and biomass as the percentage of improved pasture in a catchment increased (Quinn and Hickey 1990). Therefore, reporting on the broader management context of the landscape is important for comparing results between studies.

### 6.2 Ecological Considerations

This meta‐analysis found no significant effects of moderators such as riparian vegetation, livestock type, or study scale on aquatic insect abundance, which was unexpected given the extensive literature documenting the importance of these factors. Despite more reliable reporting within publications, important ecological factors were challenging to capture and standardize between studies and may have been generalized to an extreme degree.

For example, despite attempts to distinguish between catchment‐wide and localized, site‐level studies for all publications, these categories were often assigned based on educated assumptions when not explicitly stated within the publication. While this may have been too broad to capture potential impacts, the distinction between catchment‐level and site‐level disturbances is critical. While local exclusion of cattle from waterbodies can aid vegetation regrowth and bank stability in the immediate area, the presence of cattle within the wider drainage basin can still impact downstream aquatic communities in lotic waterbodies via runoff of excess nutrients and sediment (Weijters et al. 2009; Herbst et al. 2012; Reid et al. 2019). As a testament to this, Herbst et al. (2012) reported a significantly larger increase in EPT abundance and richness when cattle were removed at a catchment scale, compared to removal from a local, stream reach scale.

Other ecological factors, which were occasionally required to be pooled together into one sample to obtain error estimates, had significant effects on aquatic insect communities in the original studies, regardless of whether the sites were grazed or ungrazed. Such ecological factors included seasonality; for example, Conroy et al. (2016) found more pronounced effects on aquatic insect communities during autumn in Ireland due to lower stream flow and increased cattle congregation around water sources. Meanwhile, other studies reported significant differences in aquatic macroinvertebrate abundance within different stream habitats such as riffles and pools (Lorion and Kennedy 2009). Unfortunately, these additional ecological variables were not able to be considered within this meta‐analysis, although it is important to recognize their impacts on aquatic ecosystems and insect communities.

The complexity of aquatic systems, including potential upstream effects, adds another layer of difficulty to interpreting site‐level data. While many studies carefully controlled for upstream disturbances by placing control sites upstream of treatment sites, it is unrealistic to expect authors to report all potential upstream disturbances and pollutant sources that may affect the outcome of their sampling. This further highlights the variability between studies and the challenges of capturing and reporting such ecological variables across different research contexts.

### 6.3 Data Gaps and Geographic Limitations

Finally, a significant gap in this meta‐analysis arises from our attempt to isolate the impacts of livestock farming and ranching on aquatic insects. Understanding the impact of individual threats is an important first step in quantifying insect population change, but excluding studies which grouped livestock into the broader land‐use class of “agriculture” excluded data from mixed crop‐livestock farming systems, which were more common in Africa and Asia. This perpetuates existing geographical knowledge gaps in conservation and biodiversity research (Hughes et al. 2021). Further research on the impacts of other types of human disturbances, specifically those in mixed systems, is crucial to increase coverage of global studies and examine biodiversity trends in a rapidly changing world.

## 7 Conclusion

We have quantified the global impacts of livestock farming and ranching on aquatic insects from 1992 through 2023, where possible taking into account the complex nature of aquatic habitats and variable farming practices. While significant reductions in species richness were associated with livestock presence, there was little difference in total or Order‐specific abundance. This suggests that more detailed changes in community composition may be occurring, but could also reflect the challenge of consolidating data across diverse ecological contexts and management practices. Nonetheless, limiting livestock access to riparian areas is likely to benefit freshwater insect biodiversity. To improve our collective knowledge and aid future global syntheses, we call for further research, in particular reporting species richness or diversity, as our analyses were limited by a lack of studies on these metrics. Future research should also include more detailed and consistent reporting of livestock management practices, water access, and stocking densities to facilitate greater understanding of factors which create nuance in aquatic insect responses to the presence of livestock. We especially stress the need for detailed reporting on numerical stocking density to ensure repeatable and comparable levels of livestock exposure across studies. Additionally, and if time and money permit, examining abundance trends for aquatic insects at a finer taxonomic resolution is recommended, as it may provide more robust assessments of the impacts of livestock and other anthropogenic disturbances on biodiversity. Addressing these gaps will be crucial for advancing our understanding of the global and regional impacts of livestock on freshwater biodiversity and our ability to mitigate them.

## Author Contributions

**Lindsey A. Barnes:** conceptualization, data curation, formal analysis, investigation, methodology, validation, visualization, writing – original draft, writing – review and editing. **Emily Wenban‐Smith:** validation, writing – review and editing. **Grace Skinner:** methodology, software, writing – review and editing. **Lynn V. Dicks:** conceptualization, funding acquisition, supervision, writing – review and editing. **Joseph Millard:** conceptualization, methodology, project administration, supervision, validation, writing – review and editing. **Andrew J. Bladon:** conceptualization, methodology, project administration, supervision, validation, writing – original draft, writing – review and editing.

## Conflicts of Interest

The authors declare no conflicts of interest.

## Supporting information


**Data S1:** gcb70513‐sup‐0001‐Supinfo.pdf.

## Data Availability

The data that support the findings of this study are openly available in Zenodo at https://doi.org/10.5281/zenodo.16923452.

## References

[gcb70513-bib-0001] Afonso, J. , R. Ramirez‐Campillo , F. M. Clemente , F. C. Büttner , and R. Andrade . 2024. “The Perils of Misinterpreting and Misusing “Publication Bias” in Meta‐Analyses: An Education Review on Funnel Plot‐Based Methods.” Sports Medicine 54: 257–269. 10.1007/s40279-023-01927-9. 37684502 PMC10933152

[gcb70513-bib-0003] Baranov, V. , J. Jourdan , F. Pilotto , R. Wagner , and P. Haase . 2020. “Complex and Nonlinear Climate‐Driven Changes in Freshwater Insect Communities Over 42 Years.” Conservation Biology 34: 1241–1251. 10.1111/cobi.13477. 32022305

[gcb70513-bib-0004] Barnes, L. A. , and A. J. Bladon . 2023. Meta‐Analysis on the Global Impact of Livestock Farming and Ranching on Freshwater Insects (Trichoptera, Ephemeroptera, Odonata, Megaloptera, Plecoptera). OSF. https://osf.io/e39fz.

[gcb70513-bib-0005] Bartoń, K. 2024. “MuMIn: Multi‐Model Inference. Tools for Model Selection and Model Averaging With Support for a Wide Range of Statistical Models.” CRAN. 10.32614/CRAN.package.MuMIn.

[gcb70513-bib-0006] Beermann, A. J. , V. Elbrecht , S. Karnatz , et al. 2018. “Multiple‐Stressor Effects on Stream Macroinvertebrate Communities: A Mesocosm Experiment Manipulating Salinity, Fine Sediment and Flow Velocity.” Science of the Total Environment 610: 961–971. 10.1016/j.scitotenv.2017.08.084. 28830056

[gcb70513-bib-0007] Bilotta, G. S. , R. E. Brazier , and P. M. Haygarth . 2007. “The Impacts of Grazing Animals on the Quality of Soils, Vegetation, and Surface Waters in Intensively Managed Grasslands.” In Advances in Agronomy, edited by D. L. Sparks , 237–280. Academic Press.

[gcb70513-bib-0008] Bladon, A. J. , C. L. Outhwaite , R. Cooke , et al. in prep. “What's Driving Insect Decline? Top‐Down Expert Elicitation Provides a Robust Approach for Generating Taxon‐Wide Threat Rankings for a Data Deficient and Hyper‐Diverse Taxon.”

[gcb70513-bib-0009] Braccia, A. , and J. R. Voshell . 2007. “Benthic Macroinvertebrate Responses to Increasing Levels of Cattle Grazing in Blue Ridge Mountain Streams, Virginia, USA.” Environmental Monitoring and Assessment 131: 185–200. 10.1007/s10661-006-9467-3. 17171273

[gcb70513-bib-0010] Brand, C. , and M. L. Miserendino . 2015. “Testing the Performance of Macroinvertebrate Metrics as Indicators of Changes in Biodiversity After Pasture Conversion in Patagonian Mountain Streams.” Water, Air, and Soil Pollution 226: 370. 10.1007/s11270-015-2633-x.

[gcb70513-bib-0012] Ceilley, D. W. , G. G. I. I. Buckner , J. R. Schmid , and B. W. Smith . 2005. “A Survey of the Effects of Invasive Exotic Vegetation on Wetland Functions: Aquatic Fauna and Wildlife.” Final Report Prepared for the Charlotte Harbor National Estuary Program: Fort Myers, Florida.

[gcb70513-bib-0013] Clarke, A. , P. S. Lake , and D. J. O'Dowd . 2004. “Ecological Impacts on Aquatic Macroinvertebrates Following Upland Stream Invasion by a Ponded Pasture Grass (*Glyceria maxima*) in Southern Australia.” Marine and Freshwater Research 55: 709–713. 10.1071/MF04043.

[gcb70513-bib-0014] Collen, B. , M. Böhm , R. Kemp , and J. E. M. Baillie . 2012. Spineless: Status and Trends of the World's Invertebrates. Zoological Society of London.

[gcb70513-bib-0017] Conroy, E. , J. N. Turner , A. Rymszewicz , et al. 2016. “The Impact of Cattle Access on Ecological Water Quality in Streams: Examples From Agricultural Catchments Within Ireland.” Science of the Total Environment 547: 17–29. 10.1016/j.scitotenv.2015.12.120. 26780128

[gcb70513-bib-0018] Cook, R. D. 1977. “Detection of Influential Observation in Linear Regression.” Technometrics 19: 15–18. 10.1080/00401706.1977.10489493.

[gcb70513-bib-0019] Cooke, R. , C. L. Outhwaite , A. J. Bladon , et al. 2025. “Integrating Multiple Evidence Streams to Understand Insect Biodiversity Change.” Science 388: 6742. 10.1126/science.adq2110. 40179198

[gcb70513-bib-0020] Das, J. , and J. Maity . 2021. “Aquatic Entomofauna as Biological Indicator of Water Quality: A Review.” International Journal of Entomology Research 6: 257–262.

[gcb70513-bib-0022] Davidson, K. E. , M. S. Fowler , M. W. Skov , S. H. Doerr , N. Beaumont , and J. N. Griffin . 2017. “Livestock Grazing Alters Multiple Ecosystem Properties and Services in Salt Marshes: A Meta‐Analysis.” Journal of Applied Ecology 54: 1395–1405. 10.1111/1365-2664.12892.

[gcb70513-bib-0023] de Paz, V. , L. Baños‐Picón , N. Rosas‐Ramos , E. Tobajas , J. Tormos , and J. D. Asís . 2020. “The Role of Artificial Ponds in Maintaining Dragonfly Populations in an Intensified Farmland Landscape. A Case of Study in Zamora, Spain.” Journal of Natural History 54: 2439–2454. 10.1080/00222933.2020.1850901.

[gcb70513-bib-0024] Deeks, J. J. , J. P. T. Higgins , D. G. Altman , J. E. McKenzie , and A. A. Veroniki . 2013. “Chapter 10: Analysing Data and Undertaking Meta‐Analyses.” In Cochrane Handbook for Systematic Reviews of Interventions Version 6.5. Cochrane, edited by J. P. T. Higgins , J. Thomas , J. Chandler , M. Cumpston , T. Li , M. J. Page , and V. A. Welch . Cochrane. https://www.training.cochrane.org/handbook.

[gcb70513-bib-0025] DeepL . 2024. “DeepL Translator.” https://www.deepl.com/en/translator.

[gcb70513-bib-0026] del Rosario, R. B. , E. A. Betts , and V. H. Resh . 2002. “Cow Manure in Headwater Streams: Tracing Aquatic Insect Responses to Organic Enrichment.” Journal of the North American Benthological Society 21: 278–289. 10.2307/1468415.

[gcb70513-bib-0027] Dettenmaier, S. J. , T. A. Messmer , T. J. Hovick , and D. K. Dahlgren . 2017. “Effects of Livestock Grazing on Rangeland Biodiversity: A Meta‐Analysis of Grouse Populations.” Ecology and Evolution 7: 7620–7627. 10.1002/ece3.3287. 29043019 PMC5632623

[gcb70513-bib-0028] Egger, M. , G. D. Smith , M. Schneider , and C. Minder . 1997. “Bias in Meta‐Analysis Detected by a Simple, Graphical Test.” BMJ 315: 629–634. 10.1136/bmj.315.7109.629. 9310563 PMC2127453

[gcb70513-bib-0029] Epele, L. B. , and M. L. Miserendino . 2015. “Environmental Quality and Aquatic Invertebrate Metrics Relationships at Patagonian Wetlands Subjected to Livestock Grazing Pressures.” PLoS One 10: e0137873. 10.1371/journal.pone.0137873. 26448652 PMC4598092

[gcb70513-bib-0030] Eriksen, T. E. , J. E. Brittain , G. Søli , D. Jacobsen , P. Goethals , and N. Friberg . 2021. “A Global Perspective on the Application of Riverine Macroinvertebrates as Biological Indicators in Africa, South‐Central America, Mexico and Southern Asia.” Ecological Indicators 126: 107609. 10.1016/j.ecolind.2021.107609.

[gcb70513-bib-0031] Faria, A. P. J. , C. K. S. Paiva , L. B. Calvão , G. M. Cruz , and L. Juen . 2021. “Response of Aquatic Insects to an Environmental Gradient in Amazonian Streams.” Environmental Monitoring and Assessment 193: 763. 10.1007/s10661-021-09553-6. 34729664

[gcb70513-bib-0032] Felton, A. , E. Knight , J. Wood , C. Zammit , and D. Lindenmayer . 2010. “A Meta‐Analysis of Fauna and Flora Species Richness and Abundance in Plantations and Pasture Lands.” Biological Conservation 143: 545–554. 10.1016/j.biocon.2009.11.030.

[gcb70513-bib-0035] Grames, E. , A. Stillman , M. W. Tingley , and C. Elphick . 2019. “Litsearchr: Automated Search Term Selection and Search Strategy for Systematic Reviews.” R Package Version 0.2. 10.5281/zenodo.3487340.

[gcb70513-bib-0036] Gurevitch, J. , J. Koricheva , S. Nakagawa , and G. Stewart . 2018. “Meta‐Analysis and the Science of Research Synthesis.” Nature 555: 175–182. 10.1038/nature25753. 29517004

[gcb70513-bib-0037] Haase, P. , D. E. Bowler , N. J. Baker , et al. 2023. “The Recovery of European Freshwater Biodiversity Has Come to a Halt.” Nature 620: 582–588. 10.1038/s41586-023-06400-1. 37558875 PMC10432276

[gcb70513-bib-0040] Harrell, F. E., Jr. , K. L. Lee , and D. B. Mark . 1996. “Multivariable Prognostic Models: Issues in Developing Models, Evaluating Assumptions and Adequacy, and Measuring and Reducing Errors.” Statistics in Medicine 15, no. 4: 361–387. 10.1002/(SICI)1097-0258(19960229)15:4<361::AID-SIM168>3.0.CO;2-4. 8668867

[gcb70513-bib-0041] Hedges, L. V. 1981. “Distribution Theory for Glass's Estimator of Effect Size and Related Estimators.” Journal of Educational Statistics 6, no. 2: 107–128. 10.2307/1164588.

[gcb70513-bib-0043] Herbst, D. B. , M. T. Bogan , S. K. Roll , and H. D. Safford . 2012. “Effects of Livestock Exclusion on In‐Stream Habitat and Benthic Invertebrate Assemblages in Montane Streams.” Freshwater Biology 57, no. 1: 204–217. 10.1111/j.1365-2427.2011.02706.x.

[gcb70513-bib-0044] Holmes, R. , D. G. Armanini , and A. G. Yates . 2016. “Effects of Best Management Practice on Ecological Condition: Does Location Matter?” Environmental Management 57, no. 6: 1062–1076. 10.1007/s00267-016-0662-x. 26787015

[gcb70513-bib-0045] Huaranca, J. C. , A. J. Novaro , and C. E. Valdivia . 2022. “Effects of Livestock Grazing on Biodiversity: A Meta‐Analysis on Three Trophic Levels.” Journal for Nature Conservation 66: 126126. 10.1016/j.jnc.2021.126126.

[gcb70513-bib-0046] Hughes, A. C. , M. C. Orr , K. Ma , et al. 2021. “Sampling Biases Shape Our View of the Natural World.” Ecography 44, no. 9: 1259–1269. 10.1111/ecog.05926.

[gcb70513-bib-0047] IPBES . 2019. “Global Assessment Report on Biodiversity and Ecosystem Services of the Intergovernmental Science‐Policy Platform on Biodiversity and Ecosystem Services.” IPBES Secretariat.

[gcb70513-bib-0048] IUCN . 2021. “Dragonflies Threatened as Wetlands Around the World Disappear—IUCN Red List.” https://iucn.org/news/species/202112/dragonflies‐threatened‐wetlands‐around‐world‐disappear‐iucn‐red‐list.

[gcb70513-bib-0049] IUCN . 2024. “The IUCN Red List of Threatened Species.” https://www.iucnredlist.org.

[gcb70513-bib-0052] Kaboré, I. , O. Moog , M. Alp , et al. 2016. “Using Macroinvertebrates for Ecosystem Health Assessment in Semi‐Arid Streams of Burkina Faso.” Hydrobiologia 766, no. 1: 57–74. 10.1007/s10750-015-2443-6.

[gcb70513-bib-0053] Krall, M. , and P. Roni . 2023. “Effects of Livestock Exclusion on Stream Habitat and Aquatic Biota: A Review and Recommendations for Implementation and Monitoring.” North American Journal of Fisheries Management 43, no. 2: 476–504. 10.1002/nafm.10863.

[gcb70513-bib-0054] Lai, L. , and S. Kumar . 2020. “A Global Meta‐Analysis of Livestock Grazing Impacts on Soil Properties.” PLoS One 15: e0236638. 10.1371/journal.pone.0236638. 32764754 PMC7413490

[gcb70513-bib-0055] Larson, D. M. , W. K. Dodds , M. R. Whiles , J. N. Fulgoni , and T. R. Thompson . 2016. “A Before‐And‐After Assessment of Patch‐Burn Grazing and Riparian Fencing Along Headwater Streams.” Journal of Applied Ecology 53: 1543–1553. 10.1111/1365-2664.12692.

[gcb70513-bib-0056] Li, B. V. , and B. Jiang . 2021. “Responses of Forest Structure, Functions, and Biodiversity to Livestock Disturbances: A Global Meta‐Analysis.” Global Change Biology 27: 4745–4757. 10.1111/gcb.15781. 34322964

[gcb70513-bib-0058] Lorion, C. M. , and B. P. Kennedy . 2009. “Relationships Between Deforestation, Riparian Forest Buffers and Benthic Macroinvertebrates in Neotropical Headwater Streams.” Freshwater Biology 54: 165–180. 10.1111/j.1365-2427.2008.02092.x.

[gcb70513-bib-0059] Matthaei, C. D. , F. Weller , D. W. Kelly , and C. R. Townsend . 2006. “Impacts of Fine Sediment Addition to Tussock, Pasture, Dairy and Deer Farming Streams in New Zealand.” Freshwater Biology 51: 2154–2172. 10.1111/j.1365-2427.2006.01643.x.

[gcb70513-bib-0060] Maxwell, S. L. , R. A. Fuller , T. M. Brooks , and J. E. M. Watson . 2016. “Biodiversity: The Ravages of Guns, Nets and Bulldozers.” Nature 536: 7615. 10.1038/536143a. 27510207

[gcb70513-bib-0061] McDowell, R. W. , and R. J. Wilcock . 2008. “Water Quality and the Effects of Different Pastoral Animals.” New Zealand Veterinary Journal 56: 289–296. 10.1080/00480169.2008.36849. 19043466

[gcb70513-bib-0062] McIver, J. D. , and M. L. McInnis . 2007. “Cattle Grazing Effects on Macroinvertebrates in an Oregon Mountain Stream.” Rangeland Ecology & Management 60: 293–303. 10.2111/1551-5028(2007)60[293:CGEOMI]2.0.CO;2.

[gcb70513-bib-0063] Melo, A. S. , D. K. Niyogi , C. D. Matthaei , and C. Townsend . 2003. “Resistance, Resilience, and Patchiness of Invertebrate Assemblages in Native Tussock and Pasture Streams in New Zealand After a Hydrological Disturbance.” Canadian Journal of Fisheries and Aquatic Sciences 60: 731–739. 10.1139/f03-061.

[gcb70513-bib-0067] Millard, J. , A. Bladon , R. Cooke , L. Dicks , and G. Skinner . 2023. “A Preregistration Guidance Document for the Production and Collation of Meta‐Analyses for the GLiTRS Project (GLobal Insect Threat‐Response Synthesis).” OSF. https://osf.io/mw7xq.

[gcb70513-bib-0069] Most, M. , and D. Yates . 2022. Introduction to Animal Unit (AU) Concepts for Production of Sheep and Goats. University of Nebraska‐Lincoln Extension.

[gcb70513-bib-0070] Nakagawa, S. , and E. S. A. Santos . 2012. “Methodological Issues and Advances in Biological Meta‐Analysis.” Evolutionary Ecology 26: 1253–1274. 10.1007/s10682-012-9555-5.

[gcb70513-bib-0071] O'Brien, R. M. 2007. “A Caution Regarding Rules of Thumb for Variance Inflation Factors.” Quality and Quantity 41: 673–690. 10.1007/s11135-006-9018-6.

[gcb70513-bib-0072] O'Callaghan, P. , M. Kelly‐Quinn , E. Jennings , et al. 2019. “The Environmental Impact of Cattle Access to Watercourses: A Review.” Journal of Environmental Quality 48: 340–351. 10.2134/jeq2018.04.0167. 30951116

[gcb70513-bib-0073] Ogle, D. , and B. Brazee . 2009. Estimating Initial Stocking Rates. United States Department of Agriculture Natural Resources Conservation Service.

[gcb70513-bib-0074] Oliveira‐ Junior, J. M. B. d. , P. De Marco , K. Dias‐Silva , et al. 2017. “Effects of Human Disturbance and Riparian Conditions on Odonata (Insecta) Assemblages in Eastern Amazon Basin Streams.” Limnologica 66: 31–39. 10.1016/j.limno.2017.04.007.

[gcb70513-bib-0078] Outhwaite, C. L. , R. D. Gregory , R. E. Chandler , B. Collen , and N. J. B. Isaac . 2020. “Complex Long‐Term Biodiversity Change Among Invertebrates, Bryophytes, and Lichens.” Nature Ecology & Evolution 4, no. 3: 384–392. 10.1038/s41559-020-1111-z. 32066888

[gcb70513-bib-0079] Page, M. J. , J. E. McKenzie , P. M. Bossuyt , et al. 2021. “The PRISMA 2020 Statement: An Updated Guideline for Reporting Systematic Reviews.” Systematic Reviews 10, no. 1: 89. 10.1186/s13643-021-01626-4. 33781348 PMC8008539

[gcb70513-bib-0080] Petersen, I. , Z. Masters , A. G. Hildrew , and S. J. Ormerod . 2004. “Dispersal of Adult Aquatic Insects in Catchments of Differing Land Use.” Journal of Applied Ecology 41, no. 5: 934–950. 10.1111/j.0021-8901.2004.00942.x.

[gcb70513-bib-0081] Poessel, S. A. , J. C. Hagar , P. K. Haggerty , and T. E. Katzner . 2020. “Removal of Cattle Grazing Correlates With Increases in Vegetation Productivity and in Abundance of Imperiled Breeding Birds.” Biological Conservation 241: 108378. 10.1016/j.biocon.2019.108378.

[gcb70513-bib-0082] Pond, G. J. 2012. “Biodiversity Loss in Appalachian Headwater Streams (Kentucky, USA): Plecoptera and Trichoptera Communities.” Hydrobiologia 679, no. 1: 97–117. 10.1007/s10750-011-0858-2.

[gcb70513-bib-0083] Pulido‐Bosch, A. , J. P. Rigol‐Sanchez , A. Vallejos , et al. 2018. “Impacts of Agricultural Irrigation on Groundwater Salinity.” Environmental Earth Sciences 77, no. 1: 197. 10.1007/s12665-018-7386-6.

[gcb70513-bib-0084] Quinn, J. M. , A. B. Cooper , R. J. Davies‐Colley , J. C. Rutherford , and R. B. Williamson . 1997. “Land Use Effects on Habitat, Water Quality, Periphyton, and Benthic Invertebrates in Waikato, New Zealand, Hill‐Country Streams.” New Zealand Journal of Marine and Freshwater Research 31, no. 4: 579–597. 10.1080/00288330.1997.9516791.

[gcb70513-bib-0085] Quinn, J. M. , and C. W. Hickey . 1990. “Characterisation and Classification of Benthic Invertebrate Communities in 88 New Zealand Rivers in Relation to Environmental Factors.” New Zealand Journal of Marine and Freshwater Research 24, no. 3: 387–409. 10.1080/00288330.1990.9516432.

[gcb70513-bib-0086] Quinn, J. M. , R. B. Williamson , R. K. Smith , and M. L. Vickers . 1992. “Effects of Riparian Grazing and Channelisation on Streams in Southland, New Zealand. 2. Benthic Invertebrates.” New Zealand Journal of Marine and Freshwater Research 26, no. 3: 259–273. 10.1080/00288330.1992.9516520.

[gcb70513-bib-0087] R Core Team . 2023. R: A Language and Environment for Statistical Computing. R Foundation for Statistical Computing.

[gcb70513-bib-0088] Reid, A. J. , A. K. Carlson , I. F. Creed , et al. 2019. “Emerging Threats and Persistent Conservation Challenges for Freshwater Biodiversity.” Biological Reviews 94, no. 3: 849–873. 10.1111/brv.12480. 30467930

[gcb70513-bib-0089] Richardson, S. W. , M. C. Wilson , J. Nishikawa , and R. S. A. Hayward . 1995. “The Well‐Built Clinical Question: A Key to Evidence‐Based Decisions.” ACP Journal Club 123: A12. 10.7326/ACPJC-1995-123-3-A12. 7582737

[gcb70513-bib-0090] Rohatgi, A. 2024. “WebPlotDigitizer.” https://apps.automeris.io/wpd4/.

[gcb70513-bib-0091] Romero, G. Q. , D. A. Moi , L. N. Nash , P. A. P. Antiqueira , R. P. Mormul , and P. Kratina . 2021. “Pervasive Decline of Subtropical Aquatic Insects Over 20 Years Driven by Water Transparency, Non‐Native Fish and Stoichiometric Imbalance.” Biology Letters 17, no. 6: 20210137. 10.1098/rsbl.2021.0137. 34102072 PMC8187010

[gcb70513-bib-0092] Rumschlag, S. L. , M. B. Mahon , D. K. Jones , et al. 2023. “Density Declines, Richness Increases, and Composition Shifts in Stream Macroinvertebrates.” Science Advances 9, no. 7: eadf4896. 10.1126/sciadv.adf4896. 37134169 PMC10156106

[gcb70513-bib-0093] Salafsky, N. , D. Salzer , A. J. Stattersfield , et al. 2008. “A Standard Lexicon for Biodiversity Conservation: Unified Classifications of Threats and Actions.” Conservation Biology 22, no. 4: 897–911. 10.1111/j.1523-1739.2008.00937.x. 18544093

[gcb70513-bib-0094] Samways, M. J. 2020. Insect Conservation: A Global Synthesis. CABI.

[gcb70513-bib-0095] Sartorello, Y. , A. Pastorino , G. Bogliani , S. Ghidotti , R. Viterbi , and C. Cerrato . 2020. “The Impact of Pastoral Activities on Animal Biodiversity in Europe: A Systematic Review and Meta‐Analysis.” Journal for Nature Conservation 56: 125863. 10.1016/j.jnc.2020.125863.

[gcb70513-bib-0096] Schürings, C. , C. K. Feld , J. Kail , and D. Hering . 2022. “Effects of Agricultural Land Use on River Biota: A Meta‐Analysis.” Environmental Sciences Europe 34: 124. 10.1186/s12302-022-00706-z.

[gcb70513-bib-0097] Scott, D. , J. W. White , D. S. Rhodes , and A. Koomen . 1994. “Invertebrate Fauna of Three Streams in Relation to Land Use in Southland, New Zealand.” New Zealand Journal of Marine and Freshwater Research 28, no. 3: 277–290. 10.1080/00288330.1994.9516615.

[gcb70513-bib-0100] Sigutová, H. , J. Sipos , and A. Dolny . 2019. “A Novel Approach Involving the Use of Odonata as Indicators of Tropical Forest Degradation: When Family Matters.” Ecological Indicators 104: 229–236. 10.1016/j.ecolind.2019.05.001.

[gcb70513-bib-0101] Skinner, G. , R. Cooke , J. Keum , et al. 2023. “Dynameta: A Dynamic Platform for Ecological Meta‐Analyses in R Shiny.” Softwarex 23: 101439. 10.1016/j.softx.2023.101439.

[gcb70513-bib-0102] Smith, B. J. , K. J. Collier , and N. J. Halliday . 2002. “Composition and Flight Periodicity of Adult Caddisflies in New Zealand Hill‐Country Catchments of Contrasting Land Use.” New Zealand Journal of Marine and Freshwater Research 36, no. 4: 863–878. 10.1080/00288330.2002.9517138.

[gcb70513-bib-0103] Steinman, A. D. , J. Conklin , P. J. Bohlen , and D. G. Uzarski . 2003. “Influence of Cattle Grazing and Pasture Land Use on Macroinvertebrate Communities in Freshwater Wetlands.” Wetlands 23, no. 4: 877–889. 10.1672/0277-5212(2003)023[0877:IOCGAP]2.0.CO;2.

[gcb70513-bib-0105] Steyerberg, E. W. , M. J. C. Eijkemans , F. E. Harrell Jr. , and J. D. F. Habbema . 2000. “Prognostic Modelling With Logistic Regression Analysis: A Comparison of Selection and Estimation Methods in Small Data Sets.” Statistics in Medicine 19, no. 8: 1059–1079. 10.1002/(sici)1097-0258(20000430)19:8<1059::aid-sim412>3.0.co;2-0. 10790680

[gcb70513-bib-0107] Strand, M. , and R. W. Merritt . 1999. “Impacts of Livestock Grazing Activities on Stream Insect Communities and the Riverine Environment.” American Entomologist 45: 13–29. 10.1093/ae/45.1.13.

[gcb70513-bib-0108] Su, J. , F. Xu , and Y. Zhang . 2023. “Grassland Biodiversity and Ecosystem Functions Benefit More From Cattle Than Sheep in Mixed Grazing: A Meta‐Analysis.” Journal of Environmental Management 337: 117769. 10.1016/j.jenvman.2023.117769. 36958283

[gcb70513-bib-0109] Suter, G. W., II , and S. M. Cormier . 2015. “Why Care About Aquatic Insects: Uses, Benefits, and Services.” Integrated Environmental Assessment and Management 11: 188–194. 10.1002/ieam.1600. 25376941

[gcb70513-bib-0111] Valentine, J. C. , T. D. Pigott , and H. R. Rothstein . 2010. “How Many Studies Do You Need?: A Primer on Statistical Power for Meta‐Analysis.” Journal of Educational and Behavioral Statistics 35: 215–247. 10.3102/1076998609346961.

[gcb70513-bib-0112] van Klink, R. , D. E. Bowler , K. B. Gongalsky , A. B. Swengel , A. Gentile , and J. M. Chase . 2020. “Meta‐Analysis Reveals Declines in Terrestrial but Increases in Freshwater Insect Abundances.” Science 368: 417–420. 10.1126/science.aax9931. 32327596

[gcb70513-bib-0113] Vidon, P. , M. A. Campbell , and M. Gray . 2008. “Unrestricted Cattle Access to Streams and Water Quality in Till Landscape of the Midwest.” Agricultural Water Management 95: 322–330. 10.1016/j.agwat.2007.10.017.

[gcb70513-bib-0114] Viechtbauer, W. 2010a. “Conducting Meta‐Analyses in R With the Metafor Package.” Journal of Statistical Software 36: 1–48. 10.18637/jss.v036.i03.

[gcb70513-bib-0115] Viechtbauer, W. 2010b. “Metafor: Some Recommended Practices.”

[gcb70513-bib-0116] Viechtbauer, W. 2021. “Model Checking in Meta‐Analysis.” In Handbook of Meta‐Analysis, 219–254. Routledge/Taylor & Francis Group.

[gcb70513-bib-0117] Viechtbauer, W. , and M. W.‐L. Cheung . 2010. “Outlier and Influence Diagnostics for Meta‐Analysis.” Research Synthesis Methods 1: 112–125. 10.1002/jrsm.11. 26061377

[gcb70513-bib-0118] Wagner, D. L. 2020. “Insect Declines in the Anthropocene.” Annual Review of Entomology 65: 457–480. 10.1146/annurev-ento-011019-025151. 31610138

[gcb70513-bib-0119] Weijters, M. J. , J. H. Janse , R. Alkemade , and J. T. A. Verhoeven . 2009. “Quantifying the Effect of Catchment Land Use and Water Nutrient Concentrations on Freshwater River and Stream Biodiversity.” Aquatic Conservation: Marine and Freshwater Ecosystems 19: 104–112. 10.1002/aqc.989.

[gcb70513-bib-0121] Yayneshet, T. , and A. C. Treydte . 2015. “A Meta‐Analysis of the Effects of Communal Livestock Grazing on Vegetation and Soils in Sub‐Saharan Africa.” Journal of Arid Environments 116: 18–24. 10.1016/j.jaridenv.2015.01.015.

[gcb70513-bib-0122] Yoshimura, M. 2012. “Effects of Forest Disturbances on Aquatic Insect Assemblages.” Entomological Science 15: 145–154. 10.1111/j.1479-8298.2011.00511.x.

[gcb70513-bib-0123] Brand, C. , and M. L. Miserendino . 2015. “Testing the Performance of Macroinvertebrate Metrics as Indicators of Changes in Biodiversity After Pasture Conversion in Patagonian Mountain Streams.” Water, Air, and Soil Pollution 226: 370. 10.1007/s11270-015-2633-x.

[gcb70513-bib-0124] Carline, R. F. , and M. C. Walsh . 2007. “Responses to Riparian Restoration in the Spring Creek Watershed, Central Pennsylvania.” Restoration Ecology 15: 731–742. 10.1111/j.1526-100X.2007.00285.x.

[gcb70513-bib-0125] Collier, K. J. , and J. M. Quinn . 2004. “Factors Affecting Distribution and Abundance of the Mayfly *Acanthophlebia cruentata* (Leptophlebiidae) in North Island, New Zealand, Streams.” New Zealand Entomologist 27: 17–28. 10.1080/00779962.2004.9722120.

[gcb70513-bib-0126] Collier, K. J. , B. J. Smith , and B. R. Baillie . 1997. “Summer Light‐Trap Catches of Adult Trichoptera in Hill‐Country Catchments of Contrasting Land Use, Waikato, New Zealand.” New Zealand Journal of Marine and Freshwater Research 31: 623–634. 10.1080/00288330.1997.9516794.

[gcb70513-bib-0127] Conroy, E. , J. N. Turner , A. Rymszewicz , et al. 2016. “The Impact of Cattle Access on Ecological Water Quality in Streams: Examples From Agricultural Catchments Within Ireland.” Science of the Total Environment 547: 17–29. 10.1016/j.scitotenv.2015.12.120. 26780128

[gcb70513-bib-0128] Dauwalter, D. C. , K. A. Fesenmyer , S. W. Miller , and T. Porter . 2018. “Response of Riparian Vegetation, Instream Habitat, and Aquatic Biota to Riparian Grazing Exclosures.” North American Journal of Fisheries Management 38: 1187–1200. 10.1002/nafm.10224.

[gcb70513-bib-0129] de Paz, V. , L. Baños‐Picón , N. Rosas‐Ramos , E. Tobajas , J. Tormos , and J. D. Asís . 2020. “The Role of Artificial Ponds in Maintaining Dragonfly Populations in an Intensified Farmland Landscape. A Case of Study in Zamora, Spain.” Journal of Natural History 54: 2439–2454. 10.1080/00222933.2020.1850901.

[gcb70513-bib-0130] Fulgoni, J. N. , M. R. Whiles , W. K. Dodds , D. M. Larson , K. E. Jackson , and B. P. Grudzinski . 2020. “Responses and Resilience of Tallgrass Prairie Streams to Patch‐Burn Grazing.” Journal of Applied Ecology 57: 1303–1313. 10.1111/1365-2664.13623.

[gcb70513-bib-0131] García‐García, P. L. , G. Vázquez,mill , R. Novelo‐Gutiérrez , and M. E. Favila . 2017. “Effects of Land Use on Larval Odonata Assemblages in Cloud Forest Streams in Central Veracruz, Mexico.” Hydrobiologia 785: 19–33. 10.1007/s10750-016-2900-x.

[gcb70513-bib-0132] Hall, M. J. , G. P. Closs , and R. H. Riley . 2001. “Relationships Between Land Use and Stream Invertebrate Community Structure in a South Island, New Zealand, Coastal Stream Catchment.” New Zealand Journal of Marine and Freshwater Research 35, no. 3: 591–603. 10.1080/00288330.2001.9517025.

[gcb70513-bib-0133] Harner, M. J. , and K. Geluso . 2012. “Effects of Cattle Grazing on Platte River Caddisflies ( *Ironoquia plattensis* ) in Central Nebraska.” Freshwater Science 31, no. 2: 389–394. 10.1899/11-147.1.

[gcb70513-bib-0134] Hepp, L. U. , and S. Santos . 2009. “Benthic Communities of Streams Related to Different Land Uses in a Hydrographic Basin in Southern Brazil.” Environmental Monitoring and Assessment 157, no. 1–4: 305–318. 10.1007/s10661-008-0536-7. 18843547

[gcb70513-bib-0135] Herbst, D. B. , M. T. Bogan , S. K. Roll , and H. D. Safford . 2012. “Effects of Livestock Exclusion on In‐Stream Habitat and Benthic Invertebrate Assemblages in Montane Streams.” Freshwater Biology 57, no. 1: 204–217. 10.1111/j.1365-2427.2011.02706.x.

[gcb70513-bib-0136] Jun, Y.‐C. , N.‐Y. Kim , S.‐J. Kwon , et al. 2011. “Effects of Land Use on Benthic Macroinvertebrate Communities: Comparison of Two Mountain Streams in Korea.” Annales De Limnologie—International Journal of Limnology 47, no. S1: S35–S49.

[gcb70513-bib-0137] Liu, Y. , Y. Tian , Y. Gao , et al. 2022. “The Impacts of Different Anthropogenic Disturbances on Macroinvertebrate Community Structure and Functional Traits of Glacier‐Fed Streams in the Tianshan Mountains.” Water 14: 1298. 10.3390/w14081298.

[gcb70513-bib-0138] Lorion, C. M. , and B. P. Kennedy . 2009. “Relationships Between Deforestation, Riparian Forest Buffers and Benthic Macroinvertebrates in Neotropical Headwater Streams.” Freshwater Biology 54: 165–180. 10.1111/j.1365-2427.2008.02092.x.

[gcb70513-bib-0139] Matthaei, C. D. , F. Weller , D. W. Kelly , and C. R. Townsend . 2006. “Impacts of Fine Sediment Addition to Tussock, Pasture, Dairy and Deer Farming Streams in New Zealand.” Freshwater Biology 51: 2154–2172. 10.1111/j.1365-2427.2006.01643.x.

[gcb70513-bib-0140] Melo, A. S. , D. K. Niyogi , C. D. Matthaei , and C. Townsend . 2003. “Resistance, Resilience, and Patchiness of Invertebrate Assemblages in Native Tussock and Pasture Streams in New Zealand After a Hydrological Disturbance.” Canadian Journal of Fisheries and Aquatic Sciences 60: 731–739. 10.1139/f03-061.

[gcb70513-bib-0141] Mensing, D. M. , S. M. Galatowitsch , and J. R. Tester . 1998. “Anthropogenic Effects on the Biodiversity of Riparian Wetlands of a Northern Temperate Landscape.” Journal of Environmental Management 53: 349–377. 10.1006/jema.1998.0215.

[gcb70513-bib-0142] Meza‐Salazar, A. M. , G. Guevara , L. Gomes‐Dias , and C. A. Cultid‐Medina . 2020. “Density and Diversity of Macroinvertebrates in Colombian Andean Streams Impacted by Mining, Agriculture and Cattle Production.” PeerJ 8: e9619. 10.7717/peerj.9619. 32995074 PMC7501782

[gcb70513-bib-0143] O'Sullivan, M. , D. Ó hUallacháin , P. O. Antunes , E. Jennings , and M. Kelly‐Quinn . 2019. “The Impacts of Cattle Access to Headwater Streams on Hyporheic Zones.” Biology and Environment: Proceedings of the Royal Irish Academy 119B: 13–27. 10.3318/bioe.2019.02.

[gcb70513-bib-0144] O'Sullivan, M. , D. Ó hUallacháin , P. O. Antunes , et al. 2023. “Mitigation of Impacts of Cattle Access on Stream Ecosystems: Efficacy of Fencing.” River Research and Applications 40: 77–91. 10.1002/rra.4218.

[gcb70513-bib-0145] Quinn, J. M. , A. B. Cooper , R. J. Davies‐Colley , J. C. Rutherford , and R. B. Williamson . 1997. “Land Use Effects on Habitat, Water Quality, Periphyton, and Benthic Invertebrates in Waikato, New Zealand, Hill‐Country Streams.” New Zealand Journal of Marine and Freshwater Research 31, no. 4: 579–597. 10.1080/00288330.1997.9516791.

[gcb70513-bib-0146] Quinn, J. M. , R. B. Williamson , R. K. Smith , and M. L. Vickers . 1992. “Effects of Riparian Grazing and Channelisation on Streams in Southland, New Zealand. 2. Benthic Invertebrates.” New Zealand Journal of Marine and Freshwater Research 26, no. 3: 259–273. 10.1080/00288330.1992.9516520.

[gcb70513-bib-0147] Scott, D. , J. W. White , D. S. Rhodes , and A. Koomen . 1994. “Invertebrate Fauna of Three Streams in Relation to Land Use in Southland, New Zealand.” New Zealand Journal of Marine and Freshwater Research 28, no. 3: 277–290. 10.1080/00288330.1994.9516615.

[gcb70513-bib-0148] Scotti, A. , L. Füreder , T. Marsoner , U. Tappeiner , A. E. Stawinoga , and R. Bottarin . 2020. “Effects of Land Cover Type on Community Structure and Functional Traits of Alpine Stream Benthic Macroinvertebrates.” Freshwater Biology 65, no. 4: 524–539. 10.1111/fwb.13448.

[gcb70513-bib-0149] Scrimgeour, G. J. , and S. Kendall . 2003. “Effects of Livestock Grazing on Benthic Invertebrates From a Native Grassland Ecosystem.” Freshwater Biology 48, no. 3: 347–362. 10.1046/j.1365-2427.2003.00978.x.

[gcb70513-bib-0150] Smith, B. J. , K. J. Collier , and N. J. Halliday . 2002. “Composition and Flight Periodicity of Adult Caddisflies in New Zealand Hill‐Country Catchments of Contrasting Land Use.” New Zealand Journal of Marine and Freshwater Research 36, no. 4: 863–878. 10.1080/00288330.2002.9517138.

[gcb70513-bib-0151] Steinman, A. D. , J. Conklin , P. J. Bohlen , and D. G. Uzarski . 2003. “Influence of Cattle Grazing and Pasture Land Use on Macroinvertebrate Communities in Freshwater Wetlands.” Wetlands 23, no. 4: 877–889. 10.1672/0277-5212(2003)023[0877:IOCGAP]2.0.CO;2.

[gcb70513-bib-0152] Stewart, B. A. 2011. “An Assessment of the Impacts of Timber Plantations on Water Quality and Biodiversity Values of Marbellup Brook, Western Australia.” Environmental Monitoring and Assessment 173, no. 1–4: 941–953. 10.1007/s10661-010-1436-1. 20306138

[gcb70513-bib-0153] Storey, R. 2016. “Macroinvertebrate Community Responses to Duration, Intensity and Timing of Annual Dry Events in Intermittent Forested and Pasture Streams.” Aquatic Sciences 78: 395–414. 10.1007/s00027-015-0443-2.

[gcb70513-bib-0154] Uboni, C. , V. Borsato , and G. Bacaro . 2021. “Odonate Fauna Assemblages in the “Cansiglio Forest” (Insecta: Odonata).” Rendiconti Lincei. Scienze Fisiche e Naturali 32: 899–910. 10.1007/s12210-021-01029-6.

[gcb70513-bib-0155] Yadamsuren, O. , J. C. Morse , B. Hayford , J. K. Gelhaus , and P. H. Adler . 2020. “Macroinvertebrate Community Responses to Land Use: A Trait‐Based Approach for Freshwater Biomonitoring in Mongolia.” Hydrobiologia 847: 1887–1902. 10.1007/s10750-020-04220-2.

