# Supplementary material for: Differing Impacts of Livestock Farming and Ranching on Aquatic Insect Biodiversity: A Global Meta‐Analysis
Source: Glob Chang Biol. 2025 Sep 22;31(9):e70513. doi: 10.1111/gcb.70513 (PMC12452056; doi:10.1111/gcb.70513)
Supplement: Supplementary file 1 — Data S1: gcb70513‐sup‐0001‐Supinfo.pdf. [file GCB-31-e70513-s001.pdf]

FIGURES

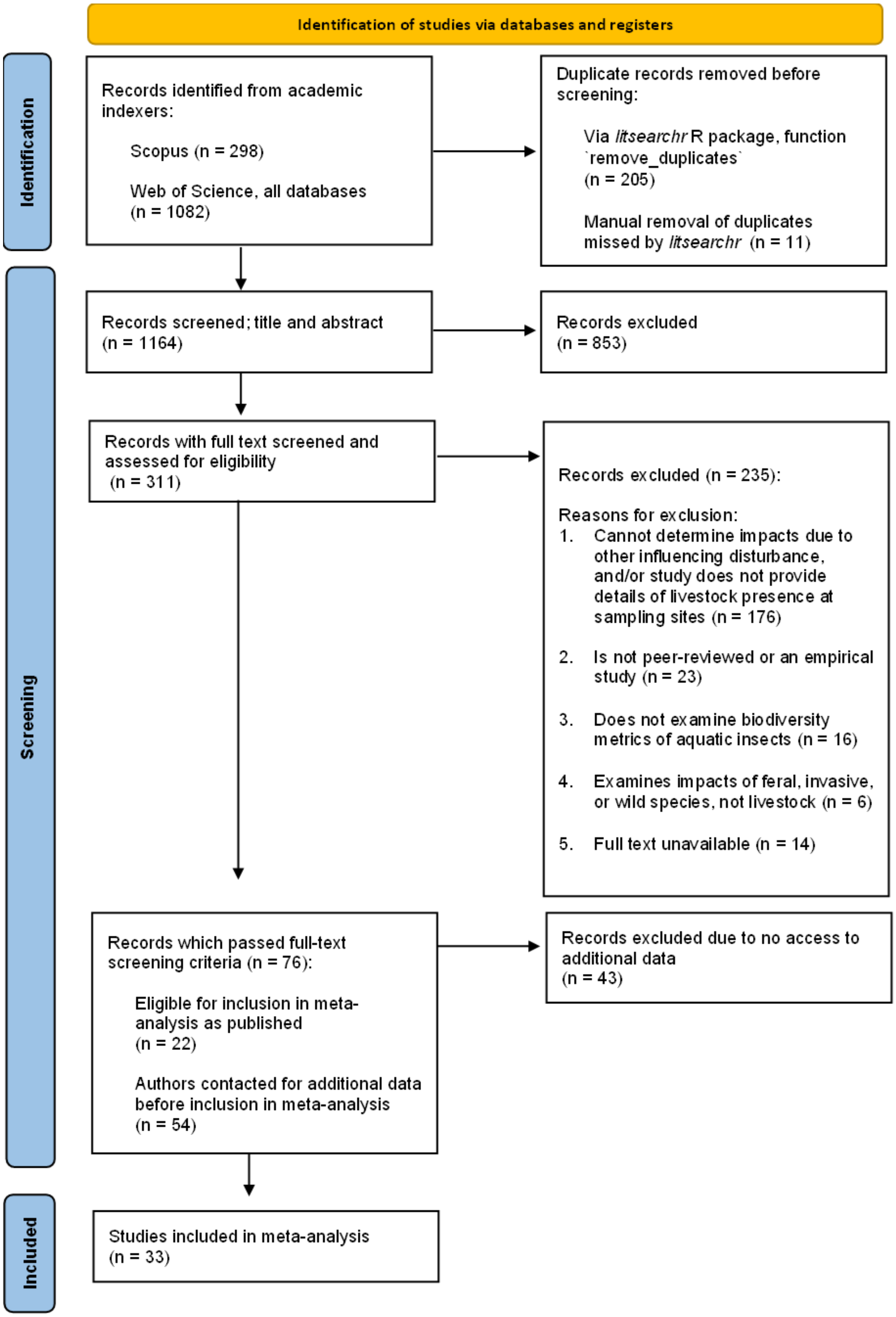

Figure S1: PRISMA (Preferred Reporting Items for Systematic Reviews and Meta-Analysis) diagram of the systematic literature selection and screening process, showing the number of studies (n) retained or excluded at each stage.

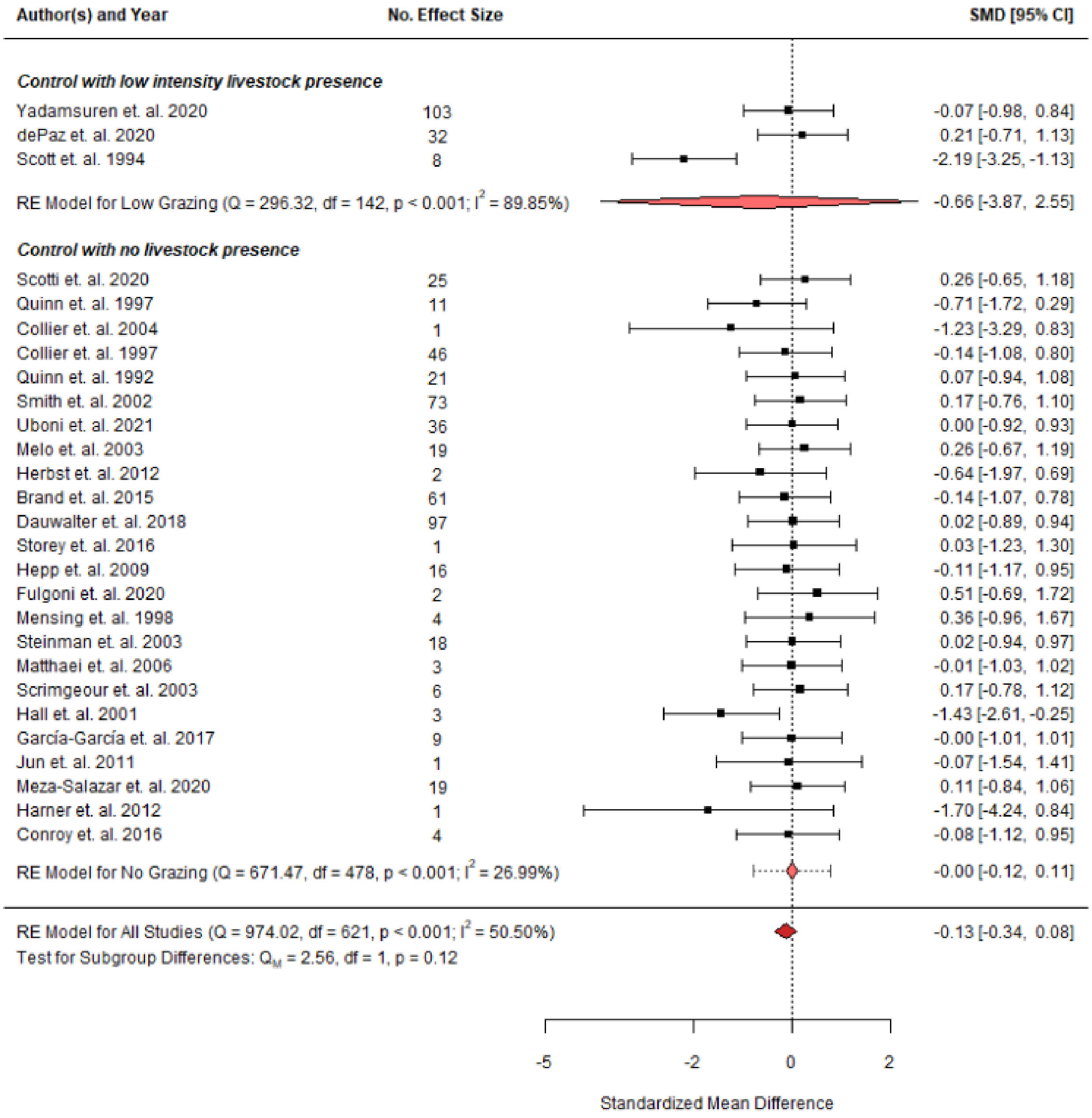

Figure S2: Forest plot of control subgroup differences test between controls with no livestock presence and controls of low livestock intensity on aquatic insect abundance.

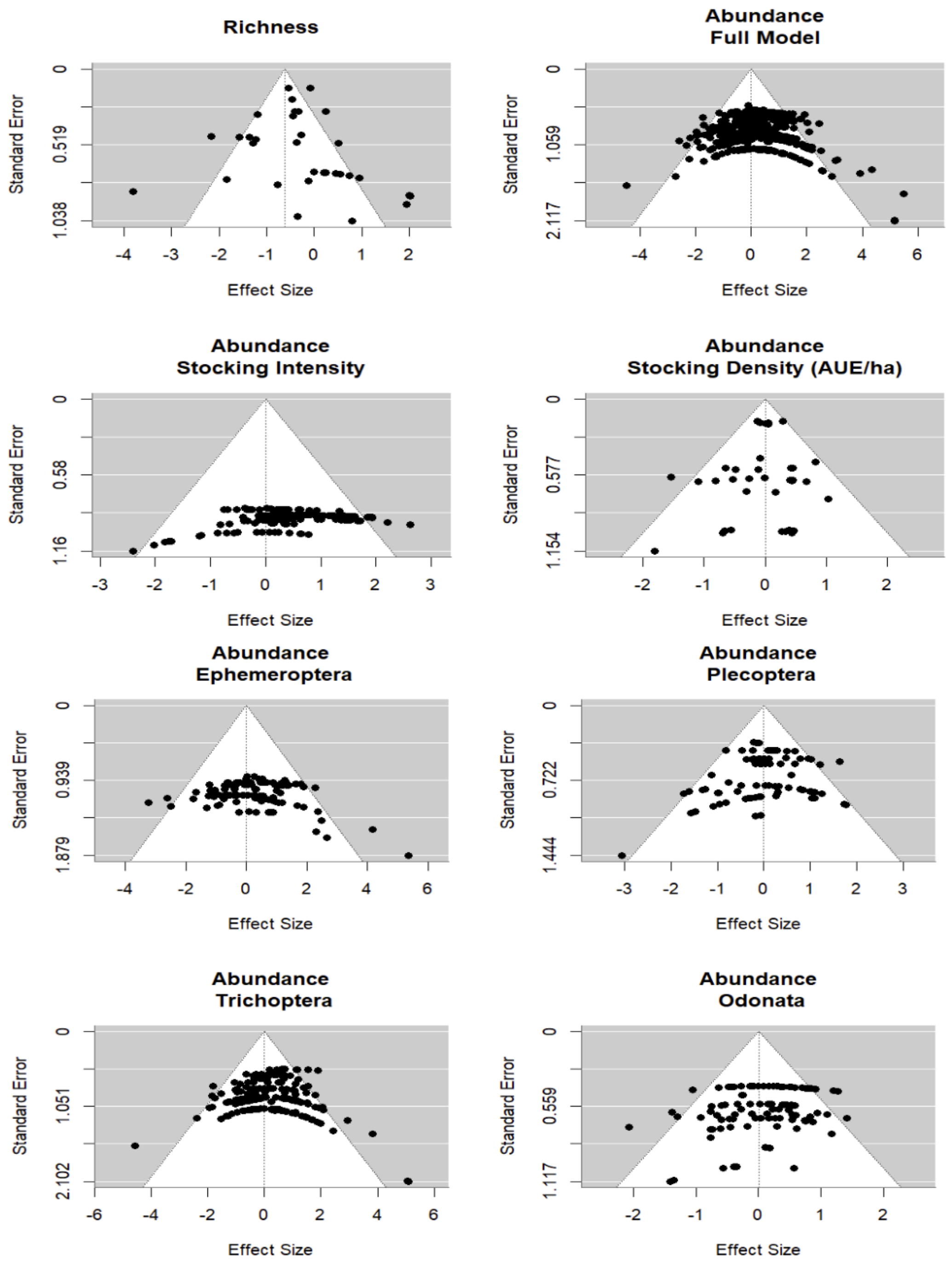

Figure S3: Funnel Plots of potential publication bias for all reported models. Shaded grey background indicates values outside of the pseudo-confidence region. Points represent individual observations.

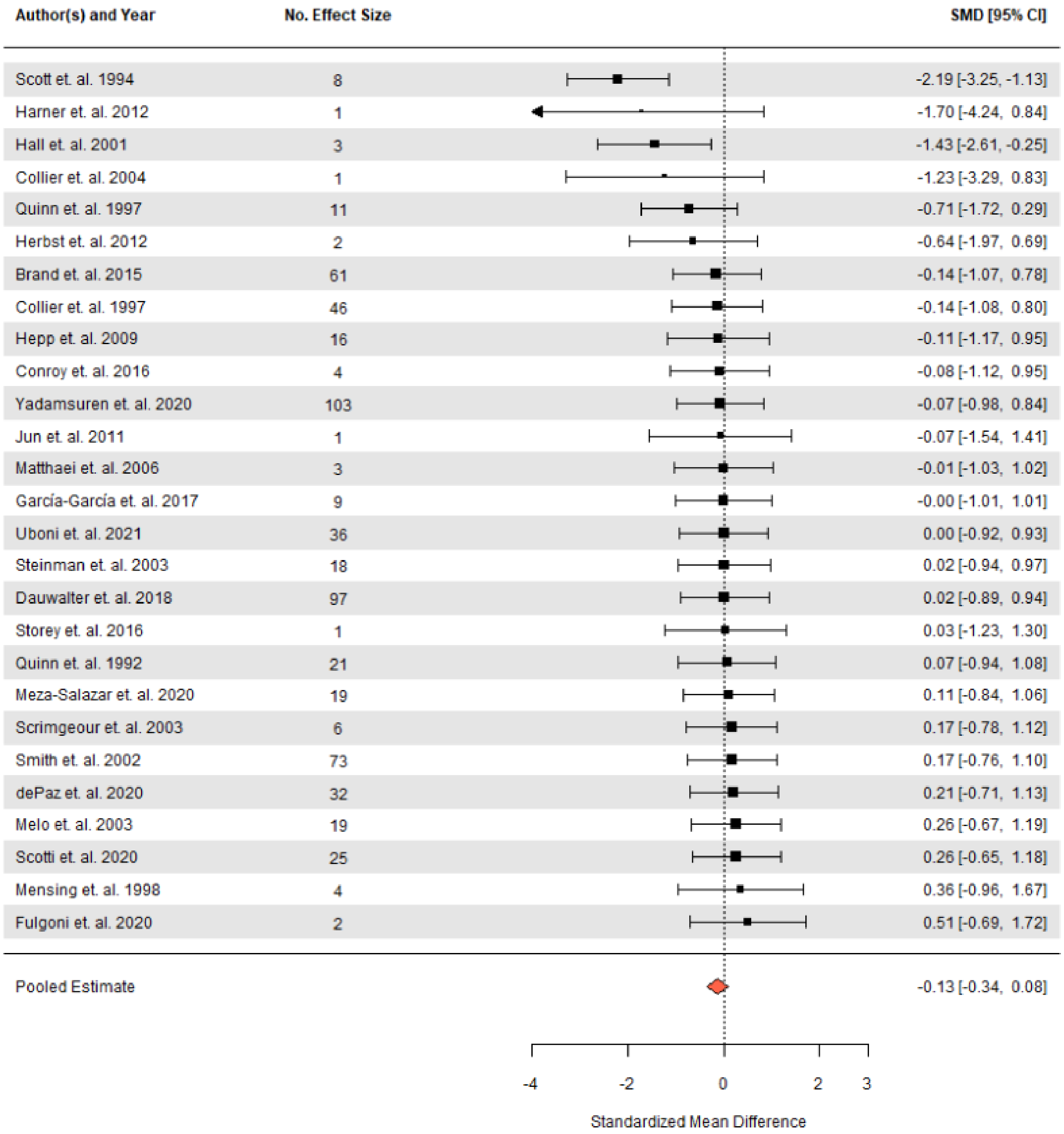

Figure S4: Forest plot of AIC_c selected multilevel model for the effect of the presence of livestock farming and ranching on the abundance of aquatic insect Orders Ephemeroptera, Plecoptera, Trichoptera, Megaloptera, Odonata, and combined EPT. Analysis was conducted on individual effect sizes (corrected standardised mean difference, Hedges' g; k = 622), however the figure shows estimates aggregated by study (n = 27). Black squares represent the pooled estimates for each study, with the size of the square indicating the number of effect sizes. Confidence intervals for each study are depicted with horizonal black lines. The overall mean estimate is depicted by the red diamond at the bottom of the plot.

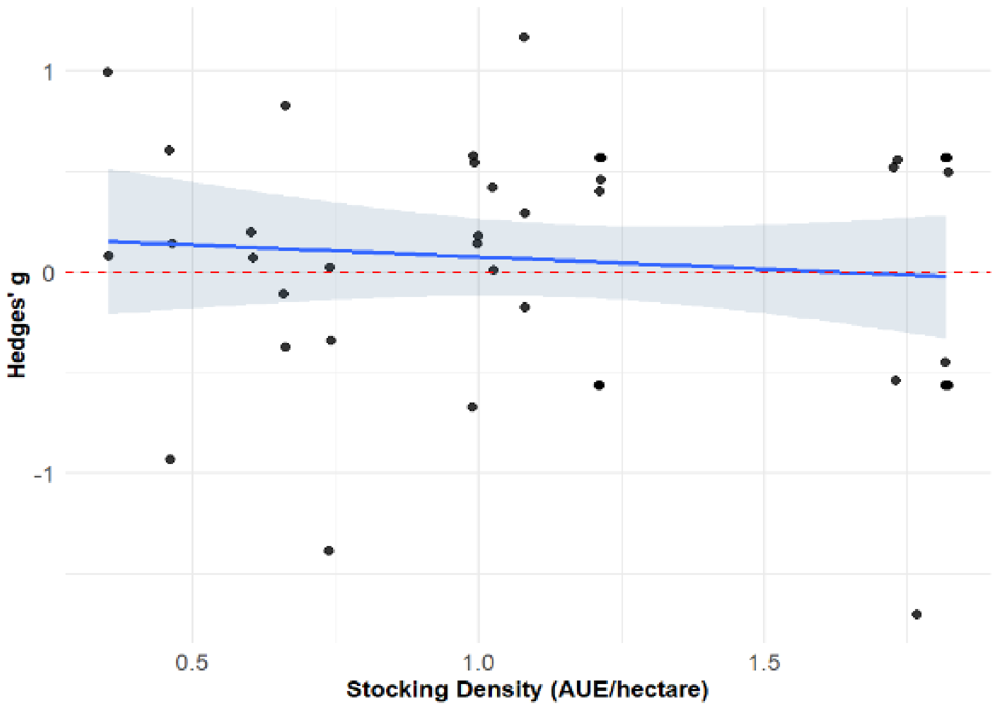

Figure S5: Regression line obtained from linear mixed effect model of livestock density (Animal Unit Equivalent (AUE)/hectare) on the abundance of aquatic insects (papers = 5, effect sizes = 48, estimate = -0.039, 95% CI [-0.427, 0.348], p = 0.839, I^2 = <0.001%). There was no effect of livestock density on aquatic insect abundance.

TABLES

Table S1: Table of final search strings used on 1 December 2023 to obtain literature results. Search strings contain the same words and search within paper title, abstract, and keywords, but are modified to fit each indexer's specific advanced search format. The number of titles returned before the removal of duplicate studies is presented.

**Academic Indexer**	**Final Search String**	**Number of titles returned**
Scopus	TITLE-ABS-KEY ((odonat* OR dragonfl* OR damselfl* OR "freshwater *invertebrate$" OR "aquatic *invertebrate$" OR "benthic *invertebrate$" OR "stream *invertebrate$" OR Trichoptera OR caddisfl* OR Ephemeroptera OR mayfl* OR Megaloptera OR dobsonfl* OR alderfl* OR Plecoptera OR stonefl* OR {EPT}) AND (livestock OR ranch* OR cattle OR bovi* OR horse$ OR sheep OR goat$ OR pasture$ OR poultry OR chicken$ OR hen$ OR pheasant$ OR ostrich OR ratite$ OR rabbit$ OR camel$ OR llama$ OR swine OR pig$ OR equine OR yak$ OR mule$ OR donkey$ OR ruminant$ OR bison OR (agricultur* W/15 "land use$")) AND (abundance OR richness OR diversity OR (structure W/2 communit*)) AND NOT (bioindicator$ OR trophic OR crop* OR genetic$))	298
Web of Science; all databases	(TS = ((odonat* OR dragonfl* OR damselfl* OR "freshwater *invertebrate$" OR "aquatic *invertebrate$" OR "benthic *invertebrate$" OR "stream *invertebrate$" OR Trichoptera OR caddisfl* OR Ephemeroptera OR mayfl* OR megalopterans OR dobsonfl* OR alderfl* OR psocoptera OR stonefl* OR "EPT") AND ((agricultur* NEAR “land use$”) OR livestock OR ranch* OR cattle OR bovi* OR horse$ OR sheep OR goat$ OR pasture$ OR poultry OR chicken$ OR hen$ OR pheasant$ OR ostrich OR ratite$ OR rabbit$ OR camel$ OR llama$ OR swine OR pig$ OR equine OR yak$ OR mule$ OR donkey$ OR ruminant$ OR bison) AND ((structure NEAR/2 communit*) OR abundance OR richness OR diversity)) NOT TS=(bioindicator$ OR trophic OR crop* OR genetic$))	1,082

Table S2: Conversion of different stocking densities reported in studies included in the meta-analysis to comparable Animal Unit Equivalents (AUE (Ogle and Brazee 2009, Most and Yates 2022)). AUE are defined by the United States Department of Agriculture Natural Resources Conservation Service (USDA-NRCS) as one AUE = 1,000 lb. (approx. 450 kg.) animal. When weights were provided, calculation of AUE was used following Most and Yates (2022):

$$^{Stocking\ density}/_{hectare} \times bodyweight(lbs) \times AUE\ adjustment = {}^{Total\ AUE}/_{hectare}$$

When weight was not reported, the closest AUE equivalent to one unit/animal of the livestock type was used to calculate AUE:

$$^{Stocking\ density}/_{hectare} \times AUE\ livestock\ equivalent = {}^{Total\ AUE}/_{hectare}$$

Study	Reported stocking density	Reported stocking density unit	Livestock type	Average body-weight (kg)	Average body-weight (lbs)	AUE adjust-ment	Total AUE per hectare
Fulgoni et al. 2020	0.825	calves per hectare	calves	193.5	426.595	0.001	**0.352**
Harner and Geluso et al. 2012	1.765	AU per hectare	adult cattle	NA	NA	0.001	**1.765**
Quinn et al. 1992	15	stock units/hectare	mixed cattle and sheep	55*	121.254	0.001	**1.819**
	10	stock units/hectare	mixed cattle and sheep	55*	121.254	0.001	**1.213**
Scrimgeour and Kendall 2003	1.33	cattle/hectare	yearling steers	349.5	770.516	0.001	**1.025**
	1.295	cattle/hectare	yearling steers	349.5	770.516	0.001	**0.998**
	0.785	cattle/hectare	yearling steers	349.5	770.516	0.001	**0.605**
Steinman et al. 2003	0.74	cow-calf pair/hectare	Braford cow-calf pairs	NA	NA	1**	**0.74**
	0.99	cow-calf pair/hectare	Braford cow-calf pairs	NA	NA	1**	**0.99**
	1.73	cow-calf pair/hectare	Braford cow-calf pairs	NA	NA	1**	**1.73**
	0.46	cow-calf pair/hectare	Braford cow-calf pairs	NA	NA	1**	**0.46**
	0.66	cow-calf pair/hectare	Braford cow-calf pairs	NA	NA	1**	**0.66**
	1.08	cow-calf pair/hectare	Braford cow-calf pairs	NA	NA	1**	**1.08**

*Study reported that 1 stock unit was equivalent to a 55kg ewe, therefore was used to convert to AUE.

** Study did not provide average bodyweight of cow-calf pairs. One cow-calf pair was assumed to be equal to one AUE, following USDA-NRCS standards (Ogle and Brazee 2009).

Table S3: Summary of all 33 publications which passed screening, from which 656 effect sizes were extracted. Asterisks (*) indicate studies which were included after authors provided additional information or raw data upon request. Taxa covered are listed at Order level or higher only: E = Ephemeroptera; P = Plecoptera; T = Trichoptera; O = Odonata; M = Megaloptera; EPT = combined Ephemeroptera, Plecoptera, and Trichoptera.

Reference	Country	No. richness effect sizes	No. abundance effect sizes	Total no. effect sizes	Taxa covered
**Brand and Miserendino 2015**	Argentina	0	61	61	E, P, T
**Carline and Walsh 2007**	United States	2	0	2	EPT
**Collier et al. 1997**	New Zealand	1	46	47	T
**Collier and Quinn 2004**	New Zealand	0	1	1	E
**Conroy et al. 2016**	Ireland	0	4	4	E
**Dauwalter et al. 2018***	United States	14	97	111	E, P, T, O
**de Paz et al. 2020***	Spain	0	32	32	O
**Fulgoni et al. 2020**	United States	0	2	2	EPT
**García-García et al. 2017**	Mexico	0	9	9	O
**Hall et al. 2001**	New Zealand	0	3	3	E, P, T
**Harner and Geluso 2012**	United States	0	1	1	T
**Hepp and Santos 2009***	Brazil	0	16	16	E, P, T, O
**Herbst et al. 2012***	United States	2	2	4	EPT
**Jun et al. 2011**	Republic of Korea	1	1	2	EPT
**Liu et al. 2022**	China	1	0	1	EPT
**Lorion and Kennedy 2009**	Costa Rica	2	0	2	EPT
**Matthaei et al. 2006**	New Zealand	3	3	6	EPT, E
**Melo et al. 2003***	New Zealand	0	13	13	E, P, T, M
**Mensing et al. 1998**	United States	0	4	4	E, T, O
**Meza-Salazar et al. 2020**	Columbia	0	19	19	E, P, T, O
**O'Sullivan et al. 2019**	Ireland	1	0	1	EPT
**O'Sullivan et al. 2023**	Ireland	1	0	1	EPT
**Quinn et al. 1992**	New Zealand	0	21	21	E, P, T
**Quinn et al. 1997**	New Zealand	1	11	12	EPT, E, T, P
**Scott et al. 1994**	New Zealand	0	8	8	E, T
**Scotti et al. 2020***	Italy	0	25	25	E, P, T
**Scrimgeour and Kendall 2003**	Canada	0	6	6	E, P
**Smith et al. 2002**	New Zealand	2	73	75	T
**Steinman et al. 2003***	United States	0	18	18	O
**Stewart 2011**	Australia	2	0	2	EPT
**Storey 2016**	New Zealand	1	1	2	EPT
**Uboni et al. 2021**	Italy	0	36	36	O

**Yadamsuren et al. 2020***	Mongolia	0	104	104	E, P, T, O, M
**TOTAL NO. EFFECT SIZES**		**34**	**622**	**656**	

Table S4: Basic model information, results of Egger's test for publication bias, and I^2 value for all models of aquatic insect diversity against livestock presence. All models were fitted with a nested random effect of ~ 1 | Study/Observation. P-values under α = 0.05, in **bold**, represent models with a likelihood of significant publication bias. Models for individual insect Orders (Ephemeroptera, Plecoptera, Trichoptera, Odonata) are all fitted to abundance data. Stocking intensity (high, medium, low, none) is a categorical variable, stocking density is a continuous variable converted to AUE (Animal Unit Equivalent). The heterogeneity statistic (I^2) is provided for each model, including results from Egger's test with z-values (z), p-values (p), limit estimates (b), and the lower 95% confidence levels (LCL) and upper 95% confidence levels (UCL) of each limit estimate.

**MODEL INFORMATION**		**EGGER'S TEST FOR PUBLICATION BIAS**					**HETEROGENEITY**
**Model name**	**Final Moderator Formula**	**z**	**p**	**b**	**LCL**	**UCL**	**I^2 (%)**
**Richness**	NA	1.714	0.086	-0.956	-1.743	-0.169	81.716
**Total abundance**	NA	-1.269	0.204	0.095	-0.062	0.253	50.504
**Ephemeroptera**	NA	0.753	0.4513	-0.091	-0.502	0.319	82.882
**Plecoptera**	~ Study scale	-2.351	**0.019**	0.375	0.067	0.683	<.0001
**Trichoptera**	NA	0.985	0.324	-0.206	-0.471	0.058	75.009
**Odonata**	NA	-2.212	**0.027**	0.555	0.100	1.010	37.066
**Stocking intensity**	~ intensity	-4.574	**< .0001**	0.871	0.491	1.251	40.170
**Stocking density**	~ density	-0.872	0.382	0.212	-0.002	0.427	17.793

Table S5: Dredge table for abundance model of all observations, with selected, least complex model within two AIC_c in **bold**. Full complex model fixed effects and interactions depicted in header: I = model intercept; LS = life stage (adult, larva); CV = control site vegetation (grassland, forest); LT = livestock type (cattle, cattle & sheep, mixed/other/unknown); SS = study scale (catchment-level, site-level); CV:LT = interaction of control site vegetation and livestock type categorical groups; CV:SS = interaction of control site vegetation and study scale categorical groups. "+" indicates presence and "NA" indicates absence of fixed effect in the model.

**I**	**LS**	**CV**	**LT**	**SS**	**CV:LT**	**CV:SS**	**df**	**logLik**	**AIC_c**	**Delta AIC_c**	**weight**
**+**	NA	+	NA	NA	NA	NA	4	-745.511	1499.087	0.000	0.214
**+**	+	+	NA	NA	NA	NA	5	-745.156	1500.410	1.324	0.111
**+**	**NA**	**NA**	**NA**	**NA**	**NA**	**NA**	**3**	**-747.278**	**1500.595**	**1.508**	**0.101**
**+**	NA	+	NA	+	NA	NA	5	-745.509	1501.115	2.028	0.078
**+**	+	NA	NA	NA	NA	NA	4	-746.848	1501.760	2.674	0.056
**+**	NA	+	+	NA	NA	NA	6	-744.817	1501.771	2.684	0.056
**+**	NA	NA	+	NA	NA	NA	5	-745.932	1501.960	2.874	0.051
**+**	NA	+	NA	+	NA	+	6	-745.099	1502.334	3.248	0.042
**+**	+	+	NA	+	NA	NA	6	-745.152	1502.440	3.353	0.040
**+**	NA	NA	NA	+	NA	NA	4	-747.240	1502.544	3.458	0.038
**+**	+	NA	+	NA	NA	NA	6	-745.473	1503.083	3.996	0.029
**+**	+	+	+	NA	NA	NA	7	-744.485	1503.153	4.066	0.028
**+**	+	+	NA	+	NA	+	7	-744.613	1503.409	4.322	0.025
**+**	+	NA	NA	+	NA	NA	5	-746.745	1503.587	4.501	0.023
**+**	NA	+	+	+	NA	NA	7	-744.811	1503.804	4.717	0.020
**+**	NA	NA	+	+	NA	NA	6	-745.930	1503.997	4.910	0.018
**+**	NA	+	+	+	NA	+	8	-744.053	1504.341	5.254	0.015
**+**	+	NA	+	+	NA	NA	7	-745.473	1505.129	6.042	0.010
**+**	+	+	+	+	NA	NA	8	-744.483	1505.201	6.114	0.010
**+**	NA	+	+	NA	+	NA	8	-744.523	1505.281	6.194	0.010
**+**	+	+	+	+	NA	+	9	-743.504	1505.301	6.215	0.010
**+**	+	+	+	NA	+	NA	9	-743.952	1506.198	7.111	0.006
**+**	NA	+	+	+	+	NA	9	-744.523	1507.340	8.253	0.003
**+**	+	+	+	+	+	NA	10	-743.950	1508.261	9.174	0.002
**+**	NA	+	+	+	+	+	10	-744.029	1508.417	9.331	0.002
**+**	+	+	+	+	+	+	11	-743.407	1509.247	10.160	0.001

Table S6: Dredge table for abundance model of Ephemeroptera observations, with selected, least complex model within two AIC_c in **bold**. Full complex model fixed effects depicted in header: I = model intercept; CV = control site vegetation (grassland, forest); SS = study scale (catchment-level, site-level). "+" indicates presence and "NA" indicates absence of fixed effect in the model.

I	CV	SS	df	logLik	AIC_c	delta AIC_c	weight
**+**	**NA**	**NA**	**3**	**-181.247**	**368.681**	**0.000**	**0.448**
**+**	NA	+	4	-180.649	369.611	0.930	0.281
**+**	+	NA	4	-181.155	370.622	1.941	0.170
**+**	+	+	5	-180.595	371.663	2.982	0.101

Table S7: Dredge table for abundance model of Trichoptera observations, with selected, least complex model within two AIC_c in **bold**. Full complex model fixed effects depicted in header: I = model intercept; LS = life stage (adult, larva); CV = control site vegetation (grassland, forest); LT = livestock type (cattle, cattle & sheep, mixed/other/unknown); SS = study scale (catchment-level, site-level); CV:SS = interaction of control site vegetation and study scale categorical groups. "+" indicates presence and "NA" indicates absence of fixed effect in the model.

I	LS	CV	LT	SS	CV:SS	df	logLik	AIC_c	delta AIC_c	weight
**+**	NA	NA	+	NA	NA	5	-342.012	694.250	0.000	0.147
**+**	**NA**	**NA**	**NA**	**NA**	**NA**	**3**	**-344.414**	**694.918**	**0.668**	**0.105**
**+**	NA	NA	+	+	NA	6	-341.328	694.973	0.723	0.102
**+**	NA	NA	NA	+	NA	4	-343.533	695.216	0.966	0.091
**+**	NA	+	NA	NA	NA	4	-343.864	695.878	1.628	0.065
**+**	+	NA	+	NA	NA	6	-341.854	696.025	1.775	0.060
**+**	NA	+	NA	+	NA	5	-342.909	696.043	1.793	0.060
**+**	NA	+	+	NA	NA	6	-341.979	696.275	2.024	0.053
**+**	+	NA	NA	NA	NA	4	-344.208	696.566	2.316	0.046
**+**	+	NA	+	+	NA	7	-341.154	696.732	2.481	0.042
**+**	+	+	NA	NA	NA	5	-343.357	696.940	2.690	0.038
**+**	NA	+	+	+	NA	7	-341.288	697.000	2.750	0.037
**+**	+	NA	NA	+	NA	5	-343.520	697.266	3.016	0.033
**+**	+	+	NA	+	NA	6	-342.769	697.855	3.605	0.024
**+**	NA	+	NA	+	+	6	-342.810	697.936	3.686	0.023
**+**	+	+	+	NA	NA	7	-341.850	698.125	3.875	0.021
**+**	NA	+	+	+	+	8	-340.878	698.304	4.054	0.019
**+**	+	+	+	+	NA	8	-341.150	698.848	4.598	0.015
**+**	+	+	NA	+	+	7	-342.588	699.601	5.351	0.010
**+**	+	+	+	+	+	9	-340.878	700.444	6.194	0.007

Table S8: Dredge table for abundance model of Odonata observations, with selected, least complex model within two AIC_c in **bold**. Full complex model fixed effects depicted in header: I = model intercept; LS = life stage (adult, larva); CV = control site vegetation (grassland, forest); SS = study scale (catchment-level, site-level). "+" indicates presence and "NA" indicates absence of fixed effect in the model.

I	LS	CV	SS	df	logLik	AIC_c	delta AIC_c	weight
**+**	+	NA	NA	4	-105.664	219.686	0.000	0.217
**+**	NA	+	+	5	-104.782	220.105	0.419	0.176
**+**	+	NA	+	5	-104.884	220.309	0.623	0.159
**+**	**NA**	**NA**	**NA**	**3**	**-107.292**	**220.797**	**1.110**	**0.125**
**+**	NA	NA	+	4	-106.428	221.213	1.527	0.101
**+**	NA	+	NA	4	-106.691	221.738	2.052	0.078
**+**	+	+	NA	5	-105.659	221.858	2.172	0.073
**+**	+	+	+	6	-104.568	221.900	2.214	0.072

Table S9: Dredge table for abundance model of Plecoptera observations, with selected, least complex model within two AIC_c in **bold**. Full complex model fixed effects depicted in header: I = model intercept; CV = control site vegetation (grassland, forest); SS = study scale (catchment-level, site-level). "+" indicates presence of fixed effect and "NA" indicates absence of fixed effect in the model.

I	CV	SS	df	logLik	AIC_c	delta AIC_c	weight
**+**	**NA**	**+**	**4**	**-91.365**	**191.195**	**0.000**	**0.630**
**+**	+	+	5	-91.166	193.038	1.843	0.251
**+**	+	NA	4	-93.724	195.912	4.717	0.060
**+**	NA	NA	3	-94.821	195.918	4.723	0.059

Table S10: Summaries of all eight final models of the impacts of livestock grazing on different aspects of aquatic insect biodiversity. Models for individual insect Orders (Ephemeroptera, Plecoptera, Trichoptera, Odonata) are all fitted to abundance data. Stocking intensity (high, medium, low, none) is a categorical variable, stocking density is a continuous variable converted to AUE (Animal Unit Equivalent). Model summaries are provided for the main effect of livestock grazing, unless specifically noted by included the *italicised* fixed effect which was also retained in the preferred model. Number of studies and observations (K) are provided, along with the overall effect size estimate, standard error (SE), t-value (T), degrees of freedom (DF), p-value (P), lower 95% confidence level (LCL), upper 95% confidence level (UCL) and heterogeneity statistic (I^2). **Bold** type indicates where the preferred model indicated a significant difference between treatment and control groups, or with fixed effects.

MODEL	NO. STUDIES	*K*	ESTIMATE	SE	*T*	DF	*P*	LCL	UCL	I^2 (%)
**Richness**	**14**	**34**	**-0.620**	**0.231**	**-2.682**	**13**	**0.019**	**-1.119**	**-0.121**	**81.716**
**Total abundance**	27	622	-0.129	0.103	-1.251	26	0.222	-0.341	0.083	50.504
**Ephemeroptera**	16	133	-0.082	0.216	-0.381	15	0.708	-0.542	0.377	75.009
**Plecoptera**	11	91	-0.381	0.181	-2.108	9	0.064	-0.791	0.028	37.066
study scale			**0.616**	**0.259**	**2.374**	**9**	**0.042**	**0.029**	**1.202**	
**Trichoptera**	15	272	-0.199	0.156	-1.274	14	0.223	-0.533	0.136	40.170
**Odonata**	9	117	0.003	0.078	0.037	8	0.971	-0.178	0.184	17.793
**Stocking intensity**	6	207	0.106	0.533	0.2	2	0.860	-2.185	2.398	82.882
high/low			-0.76	0.75	-1.014	203	0.312	-2.239	0.718	
high/none			-0.043	0.207	-0.206	203	0.837	-0.451	0.366	
medium/low			-0.72	0.752	-0.958	203	0.339	-2.203	0.762	
**Stocking density**	5	48	0.172	0.192	0.898	3	0.435	-0.438	0.783	<0.001
AUE			-0.039	0.193	-0.204	46	0.839	-0.427	0.348	
